# Human selenoprotein P and S variant mRNAs with different numbers of SECIS elements and inferences from mutant mice of the roles of multiple SECIS elements

**DOI:** 10.1098/rsob.160241

**Published:** 2016-11-23

**Authors:** Sen Wu, Marco Mariotti, Didac Santesmasses, Kristina E. Hill, Janinah Baclaocos, Estel Aparicio-Prat, Shuping Li, John Mackrill, Yuanyuan Wu, Michael T. Howard, Mario Capecchi, Roderic Guigó, Raymond F. Burk, John F. Atkins

**Affiliations:** 1 State Key Laboratory of Agrobiotechnology, College of Biological Sciences, China Agricultural University, Beijing 100193, People's Republic of China; 2 Center for Genomic Regulation, Universitat Pompeu Fabra, 08003 Barcelona, Spain; 3 Division of Gastroenterology, Hepatology, and Nutrition, Department of Medicine, Vanderbilt University School of Medicine, Nashville, TN 37232, USA; 4 Department of Biochemistry, University College Cork, Cork, Republic of Ireland; 5 Department of Physiology, University College Cork, Cork, Republic of Ireland; 6 Department of Human Genetics, University of Utah, Salt Lake City, UT 8412-5330, USA

**Keywords:** codon redefinition, ribosome specialization, selenocysteine, selenoprotein P, selenoprotein S

## Abstract

Dynamic redefinition of the 10 UGAs in human and mouse selenoprotein P (Sepp1) mRNAs to specify selenocysteine instead of termination involves two 3′ UTR structural elements (SECIS) and is regulated by selenium availability. In addition to the previously known human Sepp1 mRNA poly(A) addition site just 3′ of SECIS 2, two further sites were identified with one resulting in 10–25% of the mRNA lacking SECIS 2. To address function, mutant mice were generated with either SECIS 1 or SECIS 2 deleted or with the first UGA substituted with a serine codon. They were fed on either high or selenium-deficient diets. The mutants had very different effects on the proportions of shorter and longer product Sepp1 protein isoforms isolated from plasma, and on viability. Spatially and functionally distinctive effects of the two SECIS elements on UGA decoding were inferred. We also bioinformatically identify two selenoprotein S mRNAs with different 5′ sequences predicted to yield products with different N-termini. These results provide insights into SECIS function and mRNA processing in selenoprotein isoform diversity.

## 1. Introduction

The genetic code is not fixed as once thought but evolving, and its readout is also dynamic. Selenocysteine, Sec, specification illustrates both aspects. It is specified by UGU in *Aeromonas salmonicida*, UAG in *Blastococcus* and UGA in *Escherichia coli* and eukaryotes [1]. In the great majority of vertebrate mRNAs, UGA specifies termination and how its meaning is dynamically redefined to specify selenocysteine in a very small number of coding sequences, 25 in humans, is of great interest. While nearly all selenoprotein encoding eukaryotic mRNAs have a single UGA-specifying selenocysteine, mammalian selenoprotein P (Sepp1) mRNAs have multiple such UGAs, 10 in rat and human [2,3]. The efficiency required for independent reprogramming of the ribosome at each of such multiple UGAs raises the possibility of the ribosome involved becoming specialized for processive decoding of at least the more 3′ UGAs to specify selenocysteine [4,5]. Even in relation to certain other mRNAs, the concept of discrete classes of ribosomes has been gaining ground [6,7].

A crucial component of eukaryotic selenocysteine specification is part of the 3′ UTR forming a structure termed SECIS [8,9]. There is just one in each selenoprotein mRNA [9], except for Sepp1 mRNA which has two [2]. Eukaryotic SECIS elements are kink turn structures featuring a quartet of non-Watson Crick pairing with a central tandem of sheared G.A pairs [10] and are of two types [11,12]. The Sepp1 SECIS 2, which is closest to the 3′ end, is a form one element that lacks an additional secondary structure element in SECIS 1, a form two element. Prior work, under conditions less close to the native situation than investigated in the present work, led to the concept that SECIS 2 is primarily involved in recoding UGA 1, and SECIS 1 mediates its effect on the more 3′ UGAs [13–15].

**Figure 1.**
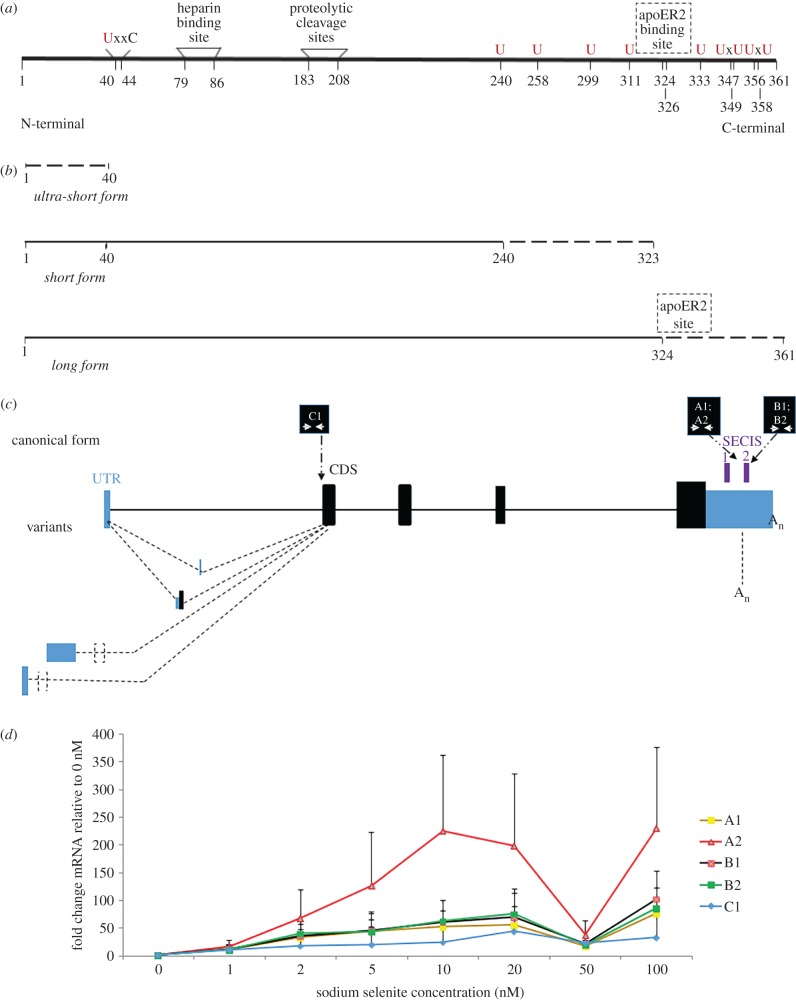
Mouse and human selenoprotein P. (*a*) Mouse Sepp1 after removal of the signal peptide. The N-terminal region includes the redox site [40–44], and heparin binding sites [79–86]. The Sec-rich C-terminal region includes the apoER2 binding site (dashed line: exact position unknown) and remaining Sec positions. U (in red) signifies sites of selenocysteine specification. (*b*) Selenoprotein P products. Ultra-short form terminates before UGA 1 at residue 40 and is undetected by the 9S4 antibody; short form progresses beyond residue 40 and does not include the apoER2 site; and long form extends beyond residue 324 and includes the apoER2 binding site. (*c*) Human selenoprotein P canonical forms and transcript variants with indication of regions that the designed qPCR primer pairs A1, A2, B1, B2 and C amplify. (*d*) Sepp1 transcript variants mRNA fold induction. Transcript quantification after normalization with GAPDH and 0 nM Se treatment. Primer pairs A1, A2, C1: long and short isoforms; Primer pairs B1, B2: long isoforms. Values are mean ± s.e.m., *n* = 3.

At least two proteins derived from the single selenoprotein P gene in mammals are present in plasma. The full-length product (P signifies plasma) [16,17] accounts for approximately 80% of plasma selenium [18,19]. The N-terminal domain, two-thirds of the 361 amino acid sequence (figure 1*a*), with its sole selenocysteine, contains a thioredoxin-like redox motif (residues 40–43) in which selenocysteine replaces one of the cysteine residues (figure 1*a*). Mass spectrometric analysis revealed that 11 shorter forms, urinary Sepp1 (Sepp1UF), have the same N-termini but their C-termini are various residues between 183 and 208 [32]. When it is ultimately filtered into urine, it is recovered by PCT cells via megalin-mediated endocytosis, preventing loss of selenium [32,33]. The redox motif has peroxidase activity when reduced by NADPH through thioredoxin reductase-1 [32]. The N-terminal domain is relevant to protection against oxidative stress, and evidence for other protective effects comes from studies on infection by *Trypanosoma congolense* [34]. Sepp1 has a heparin binding site, (residues 79–86) whose activity is dependent on acidic conditions, which, for instance, occur in inflammation.

It is unclear whether there are other functionally important Sepp1 mRNA-derived protein isoforms. The 3′ one-third of the coding sequence contains nine selenocysteine-specifying UGA codons. Products due to termination at a specific subset have been detected; in rat these are at UGA 2, 3 and 7 [35]. (This study involved ^75^Se labelling and so would not have detected termination at UGA 1.) It is unknown whether or not these derive from inefficiency in establishing the fully specialized state for selenocysteine specification and are without functional consequence. Transient transfection and *in vitro* protein synthesis studies have also shown that efficiency of selenocysteine specification at the 5′ UGAs, especially the first one, is less than can simply be obtained by substituting SECIS 2 with SECIS 1 [13–15]. Given the complexity of the processes involved, caution emanating from studies of the ionic conditions and other features of tRNA (Sec) binding to membranes [36], and the potential for events associated with expression from the endogenous gene location being relevant, we altered relevant features of the native gene in mouse, and studied the consequences at the level of product present in plasma, and phenotype evident under either selenium replete or selenium-deficient diets. Relevantly, the efficiency of selenocysteine specification for a subset of selenoprotein mRNAs varies with stress levels and in several instances is influenced by selenium levels [35,37–40]. Though not studied here, it is also pertinent that under low selenium conditions UGAs in Sepp1 mRNA undertake some level of cysteine specification [41].

The glycosylation of Sepp1 has been a complication in the analyses to date. In the rat, approximately 9000 Da of carbohydrate is present as three N-glycosylations in the N-terminal domain and, in some molecules, one O-glycosylation in the C-terminal domain [35]. Details of mouse Sepp1 glycosylation have not been reported.

In contrast to the evidence indicating occurrence and possible importance of some level of termination at least at some of the 5′-most UGAs, high efficiency of selenocysteine specification by UGAs further 3′ is evident, and when 28 UGAs occur, as in sea urchin [42], would seem to be required for synthesis of full-length product. Presumably C-terminal extension [42] was driven by the need, especially in low selenium conditions, for some tissues to have higher selenium levels than provided by an uncharacterized small molecule form(s) that lacks specificity [43,44]. Studies with controlled inactivation of mouse Sepp1 expression showed that 5–10% is expressed in non-liver tissues. However, the liver accounts for approximately 90% of plasma SEPP1 and is responsible for supplying extra-hepatic tissues with selenium through this transport mechanism [45,46]. Brain and testis [47,48] as well as bone [49], are among the important destinations. With recombinant Sepp1 lacking selenocysteine, it was shown that residues 324–326 in the selenium-rich C-terminal domain are necessary for its binding to apolipoprotein E receptor-2 (apoER2). This site is indicated with dashed lines in figure 1*a*. apoER2 binding mediates endocytosis of Sepp1, providing cells expressing this receptor with selenium for synthesis of their selenoproteins [50]. In this study, ‘long form’ refers to proteins that contain the apoER2 binding site and so can be taken up by cells. They extend beyond residue 326. We refer to ‘short forms’ as those that do not extend to amino acid 326 and do not transport selenium to tissues with the main exception of the kidney (figure 1*b*). More precise designation was hampered by the number of forms. Any ultra-short forms arising from termination at the first UGA, or before it, would not have been detected in this study as they would not contain selenium and would not contain the binding site for the monoclonal antibody, 9S4, used in these studies (the location of the binding site in the N-terminal region is unknown).

There is increasing realization of the importance of transcript variants in tunable protein synthesis [51]. This study also deals with mRNA variants derived from the single *Sepp1* gene present in humans and in mice. The mouse mRNA variants characterized to date differ in their 5′ non-coding exons, exons 1a, 1b and 1c, but have the same coding sequence (exons 2–5) [20]. All known human variants begin with the same non-coding exon, but two of the three variants have an additional exon inserted after the first exon. Suggestive of a regulatory role for the mouse variants is developmental stage changes in their distribution in heart and kidney, and localization of the 1b variant specifically to the hippocampus where its 5′ end is a target of the micro RNA Mir7 [20].

While no Sepp1 mRNA variants with different 3′ UTRs are known, such an mRNA variant is known for human selenoprotein S [21]. Selenoprotein S participates in intracellular membrane transport and consistent with being involved in removing misfolded proteins from the endoplasmic reticulum (ER), its synthesis is upregulated in conditions of ER stress [22,23]. Alternative splicing of selenoprotein S mRNA 8 nt from the start of the 3′ UTR yields a minority of transcripts that lack the sole SECIS element. Translation of these transcripts results in the penultimate codon, the sole UGA, mediating termination instead of selenocysteine specification [21]. The efficiency of selenocysteine specification and likely splicing are influenced by an mRNA structural element, known as SRE, 3′ adjacent to the UGA that in SECIS-containing transcripts specifies selenocysteine [21]. SRE elements are stem-loop structures adjacent to a subset of eukaryotic selenoprotein mRNAs that play a role in selenocysteine specification [52–55]. Following translation by the leading ribosome, the extent of proximity of the following ribosome probably influences whether it encounters a refolded SRE. Thus the efficiency of selenocysteine specification is potentially modulated by the extent of ribosome loading. On the occasions when synthesis of selenoprotein S terminates at UGA just 5′ of the full-length coding sequence terminator, the product is susceptible to degradation by the ubiquitin ligase CRL2 [38]. These findings also support the desirability of investigations with the native selenoprotein gene context.

Knowledge of Sepp1 is relevant to the recent adoption in Germany, Austria and Switzerland of the saturation of Sepp1 in plasma as a criterion for the derivation of reference values for selenium intake in adults [56], and its likely future adoption in other countries.

Further to the importance of selenoprotein P, there are intriguing aspects to how this extreme case of recoding occurs. Despite both ciliates and mammals having evolved the ability to limit selenocysteine insertion to natural positions within selenoproteins and to do so in a selenoprotein mRNA-specific manner controlled by the SECIS element in the 3′ UTR [57,58], it is still widely thought that a SECIS element will cause selenocysteine specification at any UGA in its coding sequence.

This study suggests Sepp1 SECIS functions with site specificity and further illustrates how mRNA processing may produce transcripts with altered coding potential to produce diversity in selenoprotein isoforms.

## 2. Material and methods

### 2.1. Bioinformatic discovery and quantification of alternative transcript isoforms

Alternative transcript isoforms for Sepp1 and selenoprotein S genes were searched using various data sources. One was a map of polyA sites inferred by sequencing with a protocol specifically developed for this purpose (polyA-Seq) [59]. These data span multiple tissues in five mammals (electronic supplementary material, figure S1*d*). Importantly, this sequencing protocol permits distinction of the source strand of the RNA reads. This can yield accurate strand quantification and exclude the potential confounding factor of reads coming from overlapping genes. This is relevant since the 3′ UTR of Sepp1 partially overlaps gene CCDC152 on the opposite strand, making quantification of the 3′ variants of Sepp1 practically impossible without strand information. Alternative polyA sites inferred by polyA-Seq were first inspected in the UCSC genome browser [60]. Then the raw quantification values were downloaded from the Supplementary materials of Derti *et al*. [59] and manually inspected.

Secondly, the human tissue-specific quantifications from the Genotype-Tissue Expression consortium (GTEx) [61] were considered. The expression values across human tissues of the exons and exon junctions annotated for selenoprotein S were extracted, plotted and finally compared with the polyA-seq quantification. As the RNA-seq protocol used in GTEx was not strand-specific, these data could not be used for quantification of the 3′ UTR variants in selenoprotein P. However, the GTEx data allowed us to profile the expression across human tissue variants in the rest of the gene structure (electronic supplementary material, figures S1B, S1C, S2B and S2C).

### 2.2. Bioinformatic analysis of SECIS elements

Selenoprotein P genes were identified in vertebrate genomes using the program Selenoprofiles [62]. Their SECIS elements were identified by scanning the sequences downstream of the coding sequences using SECISearch3 [63]. The SECIS sequences were aligned with t-coffee [64], and then visualized using Jalview [65]. The predicted phylogenetic tree of SECIS elements was computed based on sequence identity, using the average distance method available within Jalview (electronic supplementary material, figure S3*b*).

### 2.3. Experimental analysis of selenium supplementation in tissue culture cells

Human hepatic carcinoma cell lines (HEPG2) were cultured and selenium supplemented following a previously published protocol [66]. Total RNA and genomic DNA (gDNA) were then isolated from cells seeded at a density of around 0.8 × 10^6^ in a 60 mm cell culture dish. Three cell culture replicates were produced to generate data points. RNA was isolated as per manufacturer's protocol using RNeasy mini kit from Qiagen (74104) and gDNA was isolated as per manufacturer's guidelines using Purelink genomic DNA mini kit from Invitrogen (KI820-01). Total RNA was run on an agarose gel to determine RNA integrity. Total RNA and gDNA quantity and purity were determined by spectrophotometry using the Nanodrop 1000 from Thermo Scientific. RNA was equalized to 2 µg per reaction, treated with RQ1 Promega DNase and reverse transcribed into complementary DNA (cDNA) using Superscript III first strand synthesis system from Invitrogen (18080051). gDNA was also equalized to 2 µg per reactions. Real-time qPCR experiments were carried out by equalizing template cDNA and gDNA to 66.7 ng per reaction per well. Reactions (20 µl) were carried out in triplicate with each containing a mixture of 4 µl template, 10 µl SYBR green Fast Start Essential DNA Green Master from ROCHE (06402712001), 1 µl PCR grade water and 1 µl each of 10 µM stock forward and reverse primers. Primers were synthesized by Integrated DNA Technologies and the sequences are listed in the electronic supplementary material, table S1*a*. All reactions were performed in triplicate. Non-reverse-transcribed controls were also amplified to ensure that the RNA used was free from gDNA contamination.

Primer efficiency to target binding was determined using *T*_m_ melting peak analysis and agarose gel electrophoresis. Ct values from cDNA were normalized against Ct values of gDNA/GAPDH cDNA to compensate for variations of input RNA/cDNA and differences in reverse transcription efficiency. Relative mRNA-fold induction was calculated using the ΔΔCT method on the normalized values. Changes in expression of each transcript variants at different selenium concentration were expressed as mRNA fold induction divided by mRNA fold induction values at 0 nM added selenium concentration for each of the primer pairs.

### 2.4. Generation of *Sepp1*^ΔSECIS1^ and *Sepp1*^ΔSECIS2^ mice

**Figure 2.**
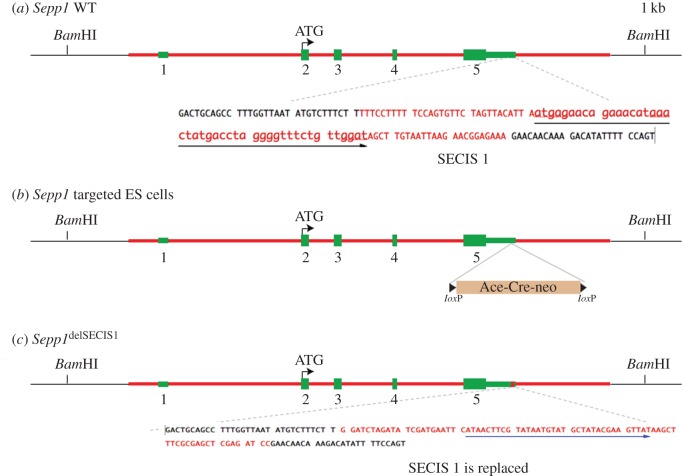
Generation of a knockout mouse line, *Sepp1*^ΔSECIS1^, that has deleted the first SECIS signal of the *Sepp1* gene. To generate the *Sepp1*^ΔSECIS1^ allele, a genomic fragment (red line) containing sequence from 5′-CTGAAGCAACAGCTAAAAGA-3′ to 5′-AACACTCCATGCAAACTACA-3′ of the *Sepp1* gene was used for constructing the targeting vector. (*a*) Genomic structure of the WT *Sepp1* gene, with its five exons shown in green. The 3′ UTR sequence is shown, with the sequence shown in red being that of SECIS 2. (*b*) In the targeting vector, a self-excising neo cassette (Ace-Cre-neo, also named ACN) was used to replace the SECIS 1 sequence. (*c*) During the cross between chimaeric males and WT females, the ACN neo cassette is deleted automatically, resulting in a clean heterozygous *Sepp1*^ΔSECIS1^ allele.

**Figure 3.**
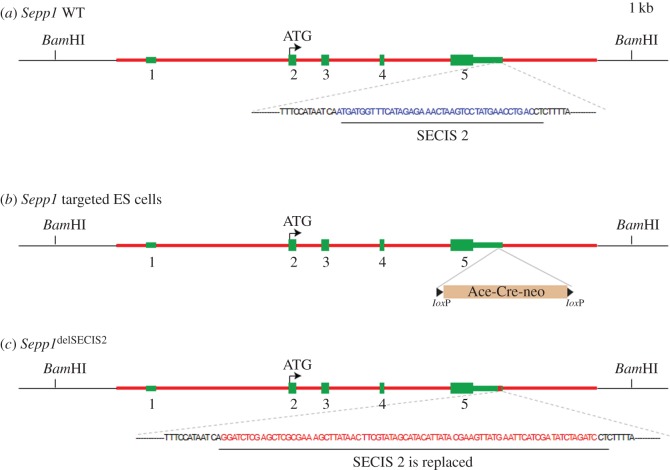
Generation of a knockout mouse line, *Sepp1*^ΔSECIS2^, that has deleted the second SECIS signal of the *Sepp1* gene. To generate the *Sepp1*^ΔSECIS2^ allele, a genomic fragment (red line) containing sequence from 5′-CTGAAGCAACAGCTAAAAGA-3′ to 5′-AACACTCCATGCAAACTACA-3′ of the *Sepp1* gene was used for constructing the targeting vector. (*a*) Genomic structure of the WT *Sepp1* gene, with its five exons shown in green. The 3′ UTR sequence is shown, with the SECIS 2 signal highlighted in blue. (*b*) In the targeting vector, a self-excising neo cassette (Ace-Cre-neo, also named ACN) was used to replace the SECIS 2 sequence. (*c*) During the cross between chimaeric males and WT females, the ACN neo cassette is deleted automatically, resulting in a clean heterozygous *Sepp1*^ΔSECIS2^ allele.

The method used was described previously [67], and construct features are illustrated in figures 2 and 3. We first used recombineering to subclone a 13.1 kb genomic fragment from a BAC clone RP23-41H17, which was obtained from the BACPAC resources (http://bacpac.chori.org/). The two oligos used in this step (electronic supplementary material, table S1*b*) were WS785 and WS786. The resulting plasmid from this step was named pStartK-Sepp1. We next designed two oligos WS1214SECIS1camF and WS1215SECIS1camR to PCR amplify pKD3 which contains a chloramphenicol resistance gene (*cat*). The PCR product was used for recombineering with the plasmid pStartK-Sepp1.

In the correctly recombined clones, the SECIS 1 sequence was now replaced by a BamHI flanked *cat*. The *cat* gene was then cut out by BamHI digestion, and a BglII flanked neo selection cassette was inserted. The resulting plasmid is named pStartK_Sepp1_dSECIS1_ACN. Similarly starting from pStartK-Sepp1 (above), we designed two oligos WS789 and WS790 (electronic supplementary material, table S1*b*) to PCR amplify pKD3 which contains a chloramphenicol resistance gene (*cat*). The PCR product was used for recombineering with the plasmid pStartK-Sepp1. In the correctly recombined clones (named pStartK-Sepp1-789), the second SECIS sequence was now replaced by a *cat* gene flanked by BamHI restriction sites. The *cat* gene was then removed by BamHI digestion, and a BglII flanked neo selection cassette (designated ACN) was inserted. The resulting plasmid was named pStartK-Sepp1-dSECIS2-ACN.

To add a negative selection HSV-tk gene, Gateway recombination is performed to quickly transfer the modified Sepp1-ACN into an HSV-tk containing vector named pWSTK6. The resulting targeting vectors are named pWSTK6-Sepp1-dSECIS1 and pWSTK2-dSepp1-dSECIS2, respectively. Standard electroporation of linearized targeting vector into ES cells was performed as described [67]. Southern blot analysis was performed to identify correctly targeted ES cell clones. The 3′ Southern probe template (301 bp) was amplified by PCR from the BAC clone RP23-41H17 with primers WS871Sepp1-3F and WS872Sepp1-3R (electronic supplementary material, table S1*b*). DNA isolated from ES cells was digested with BamHI, and run on a 0.9% agarose gel.

A Southern blot was performed with the 3′ probe. The wild-type (WT) band is 15.7 kb for both SECIS 1 and 2, and targeted mutant band is 5.1 kb for SECIS 1 and 4.6 kb for SECIS 2. The positive targets were further confirmed by long-range PCR.

### 2.5. Generation of *Sepp1*^U59S^ mice to give U40S after signal peptide removal

To construct the targeting vector for *Sepp1*^U59S^, we started from pStartK-Sepp1 (above). We used PCR-based mutagenesis to convert the DNA encoding the first selenocysteine from TGA to TCA, which now encodes serine. We then inserted the self-excising neo selection cassette ACN in the BglI site before the second exon. The resulting plasmid was named pStartK-Sepp1U59SACN. To add a negative selection HSV-tk gene, Gateway recombination was performed to quickly transfer Sepp1U59SACN into an HSV-tk containing vector named pWSTK2. The resulting targeting vector was named pWSTK2-Sepp1U59SACN. Standard electroporation of the linearized targeting vector into ES cells was performed as described [67]. Southern blot analysis was performed to identify correctly targeted ES cell clones. The 5′ Southern probe template (476 bp) was amplified by PCR from the BAC clone RP23-41H17 with primers WS869 and WS870 (table S1*b*). DNA isolated from ES cells was digested with BamHI, and run on a 0.9% agarose gel. Southern blot was done with the 5′ probe. The WT band is 15.7 kb, and targeted mutant band is 6.9 kb. The positive targets were further confirmed by Southern blot analysis with XbaI digest, 5′ probe.

### 2.6. Blastocyst injection and mouse breeding

Targeted ES cells for *Sepp1*^ΔSECIS1^, *Sepp1*^ΔSECIS2^ and *Sepp1*^U59S^ were injected into blastocysts using a standard protocol. Male chimaeric mice were bred with C57BL/6 females to obtain the desired alleles. As we used the self-excising neo cassette ACN, the neo was automatically deleted in the F_1_ generation of heterozygotes.

## 3. Results

### 3.1. Alternative transcripts for human Sepp1

To explore possible natural variants of Sepp1 mRNA with differing 3′ UTRs, we searched for alternative transcript variants of Sepp1 genes. A global map of polyA sites in multiple tissues of five mammals: human, rhesus, dog, mouse and rat [59] was first used. In all species, the major polyA site predicted for Sepp1 was ‘canonical’, located just downstream of SECIS 2. In dog, rhesus and human, a second polyA site was identified, further away. This site is predicted to result also in canonical mRNA, carrying both SECIS 1 and 2. Interestingly, we found an additional, well-supported alternative site in human and rhesus, not present in dog, mouse and rat (electronic supplementary material, figure S1*d*). This site resides in between the two SECIS elements, and thus would result in mRNAs lacking SECIS 2 (figure 1*c*). The site was observed in all human and rhesus tissue samples. Its quantification relative to the major canonical form was similar across tissues, ranging from 10 to 25% in dispersed humans, and from 0.5 to 9% in rhesus. This variant is produced in similar proportions across tissues.

Additionally, we searched the expression profiles generated for the GTEx project, derived from post-mortem samples of various human tissues [61]. Although it was not possible to quantify in these profiles the 3′ UTR variants (see Material and methods), we could obtain expression profiles for other Sepp1 mRNA variants. In particular, we observed usage of a non-coding alternative first exon located approximately 13.5 kb upstream of the first exon in the canonical form (see the electronic supplementary material, figure S1*a*). This alternative first exon was detected specifically in blood and liver, where, nevertheless, it still constitutes only a minor fraction of the total Sepp1 mRNAs, among which the canonical form predominates. The expression analysis also highlighted usage of two cassette exons located between the first and second exons of canonical Sepp1 mRNA (electronic supplementary material, figure S1*a* and figure 1*c*). The first cassette exon was detected at very low levels in all tissues. The second cassette exon was detected specifically in the small intestine, liver, kidney and transverse colon, but again only as a minor fraction of the amount of the canonical mRNA (electronic supplementary material, figures S1*b*,*c*).

To quantify the different mRNA variants upon selenium supplementation, we designed multiple primer pairs targeting either the region specific to the long variant which contains both SECIS 1 and SECIS 2 (B1 and B2), or common parts namely the long variant and the recently identified short variant lacking SECIS 2 (A1, A2 and C; electronic supplementary material, table S1 and figure 1*c*). An issue with this approach is that the 3′ UTR of *Sepp1* overlaps with another gene on the opposite strand. While A1, A2, B1 and B2 map to the overlapping region, C is upstream and thus works as a control for the A primers. The differences in transcript expression between A1/A2/C and B1/B2 primers would indicate the proportion at which the two distinct mRNAs are expressed. The HEPG2 cell line was cultured in media supplemented at different selenium concentrations. Liver was chosen since it is the tissue with the highest Sepp1 expression, and considered the major ‘exporter’ of selenium to other tissues. Ct values of the cDNA for each sample were normalized to GAPDH cDNA values. A second normalization was performed against the 0 nM added selenium values. In general, each of the primers shows a multiphasic distribution which was not reported previously. A first bell-shaped distribution of Sepp1 transcript expression was observed followed by a second point of increase at a higher selenium concentration. This finding suggests that although the different Sepp1 transcript variants are likely regulated by increasing selenium concentration, no difference in the distribution of long and short transcripts was observed (figure 1*d*).

### 3.2. Generation of mutant mice and characterization of their Sepp1

**Figure 4.**
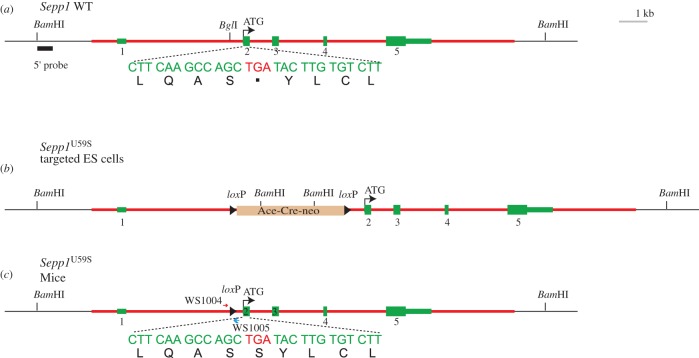
Generation of a mouse line with Ser in place of the first selenocysteine (Sec). A genomic fragment (highlighted in red) containing sequence from 5′-CTGAAGCAACAGCTAAAAGA-3′ to 5′-AACACTCCATGCAAACTACA-3′ of the *Sepp1* gene was used for constructing the targeting vector. (*a*) Genomic structure of the WT *Sepp1* gene, with its five exons in green. The second exon contains the start codon ATG. *Sepp1* has 10 selenocysteines encoded each by a UGA codon. The first of the 10 TGAs that can specify selenocysteine is located within the second exon. The remaining nine TGA sequences are located in exon 5. (*b*) After homologous recombination in the ES cells, one copy of the endogenous *Sepp1* gene is replaced by the modified sequence in the targeting vector, which has the first TGA (Sec) changed into TCA (Ser) and a self-excising neo cassette (Ace-Cre-neo, also named ACN) inserted into the *Bgl*I site between first and second exons. (*c*) During the cross between chimaeric males and WT females, the ACN neo cassette is deleted automatically, resulting in a clean heterozygous allele *Sepp1*^U59S^. U40S referred to below is after the signal peptide is removed.

Multiple selenocysteine specifying UGA codons and two SECIS elements in its 3′ UTR (instead of one) make selenoprotein P mRNA unique, with alteration of these features likely informative about the nature of the decoding and the forms of the products secreted into plasma. We generated mutant mice by the procedures described in the Material and methods section. One mutant had SECIS 1 deleted and replaced by a loxP site (figure 2), and another had SECIS 2 similarly deleted (figure 3). The third mutant had the first UGA substituted with a serine codon (figure 4). Plasma Sepp1 forms were then studied in the resulting mice.

Plasma selenium biomarkers (Sepp1 concentration, glutathione peroxidase (Gpx) activity and selenium concentration) were quantified. Over 97% of plasma selenium is present in two selenoproteins—Sepp1 (approx. 80%) and Gpx-3 (approx. 18%)—in mice fed the element as selenite [18,19]. Sepp1 was isolated from plasma using the antibody 9S4 and its selenium content was determined. In addition, it was subjected to SDS-PAGE analysis. Attempts to characterize Sepp1 forms with mass spectrometry were difficult to interpret because of the large number of forms that were present. Those results will not be reported here.

### 3.3. Mutation of the first selenocysteine to serine

The first selenocysteine residue of Sepp1 is distinguished by its location remote from the other nine residues (figure 1*a*). Because UGA 1 must be translated before the other UGAs, it was mutated to a serine codon to allow us to determine whether its presence affected other aspects of Sepp1 synthesis and secretion.

Some of the characteristics of U40S homozygous mice have been published elsewhere. They had no obvious clinical abnormalities and tolerated severe selenium deficiency without developing the neurological signs observed in homozygous Sepp1 deletion mice fed a selenium-deficient diet [68].

**Figure 5.**
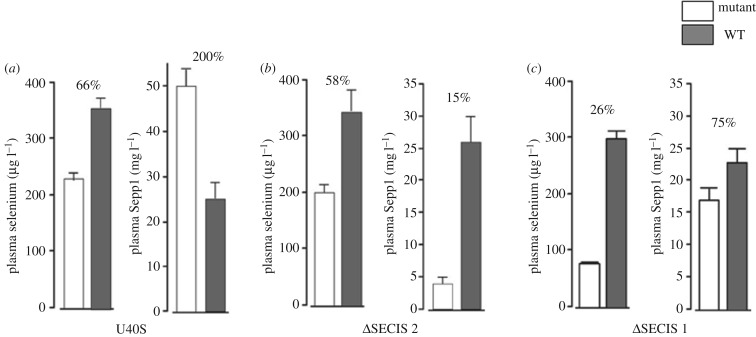
Plasma selenium biomarkers of mutant selenoprotein P gene mouse strains. (*a*) U40S; (*b*) ΔSECIS 2; (*c*) ΔSECIS 1. Values are means + 1 s.d., *n* = 5. Pairs of values with percentages above them are different (*p* < 0.05) by Student's *t*-test.

U40S homozygote mice had twice as much Sepp1 in their plasma as did congenic WT littermates but only 66% as much selenium (figure 5*a*). Gpx activity was not affected and neither were selenium levels in liver, kidney, brain, testis, quadriceps and the whole body (results not shown).

**Figure 6.**
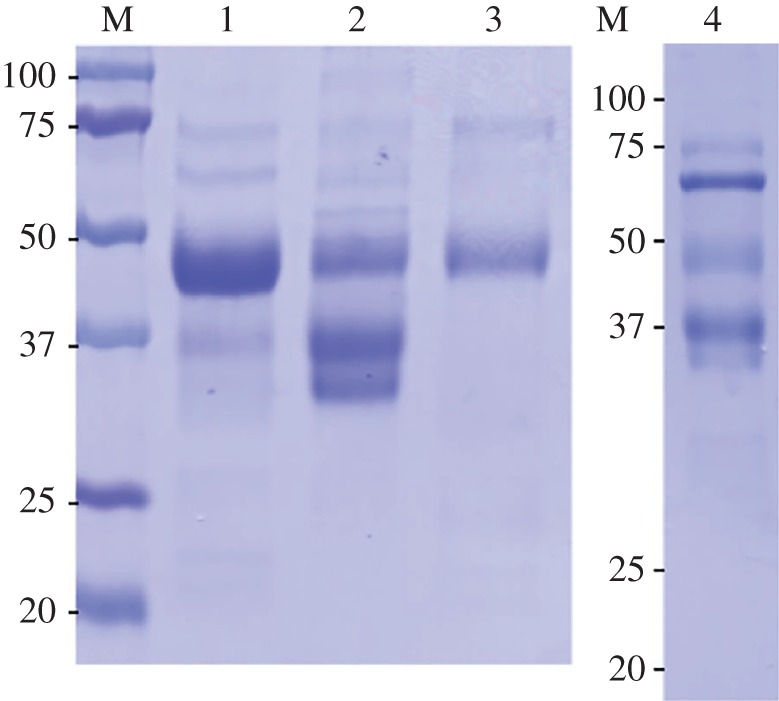
Analysis of selenoprotein P preparations from plasma of the mutant mice strains by SDS-PAGE. Numbered lanes contain preparations: lane 1, WT; lane 2, U40S; lane 3, ΔSECIS 2; lane 4, ΔSECIS 1. M indicates molecular weight markers. Plasma from each mouse was passed through a column containing a monoclonal antibody (9S4) against the N-terminal portion of Sepp1. Then a Sepp1 fraction was eluted. The amount of Sepp1 fraction that contained 2 µg of protein was loaded onto each lane.

Forms of plasma Sepp1 from homozygous U40S mice had a different SDS-PAGE migration pattern from that of WT forms. The protein from WT mice migrated as a broad band extending from approximately 50 kDa to approximately 45 kDa and a much lighter band at approximately 37 kDa (figure 6, lane 1). The predominant Sepp1 from U40S mice migrated at 37 kDa and another, also dense, product migrates even farther (figure 6, lane 2). A third, but less dense, product migrated just below 50 kDa, but above the other two products. These observations indicate that the Sepp1 forms from U40S mice plasma are, on average, smaller than those making up WT plasma Sepp1.

The purified preparation of Sepp1 from U40S mice contained an average of 1.6 selenium atoms per molecule—many fewer than the 5.8 atoms per molecule contained in the WT preparation (table 1). In a separate experiment, the selenium atoms per WT Sepp1 molecule in each of the three Sepp1-containing bands were estimated. Aliquots of a single preparation of Sepp1 were applied to four lanes of an SDS-PAGE gel. The three bands were scanned for density and cut from each lane. Selenium was determined in each band. Sepp1 in each band was estimated by multiplying the amount of Sepp1 applied to the lane by its fraction of the density of all three bands. The products—from upper to lower—were estimated to contain 2.5 ± 0.8, 0.20 ± 0.12 and 0.06 ± 0.05 selenium atoms per Sepp1 molecule, respectively. The value of 0.20 from the middle U40S mouse product is taken to imply that only a modest minority of decoding events involving the first UGA results in selenocysteine rather than an amino acid such as cysteine or serine being specified.

**Table 1.** Selenium atoms per molecule of selenoprotein P preparations from plasma of the mutant strains (WT, wild-type).

mouse strain	selenium atoms/selenoprotein P molecule^a^
WT	5.7, 5.9
U40S	1.4, 1.7
ΔSECIS 2	6.4, 8.1
ΔSECIS 1	1.0

^a^Each value is from a preparation of selenoprotein P from different mice.

These findings indicate that many of the forms of Sepp1 from U40S mice lacked some, or all, of the selenium-rich C-terminal domain and, possibly, some of their carbohydrate. It is clear, however, that some Sepp1 forms reaching to residues 324–326 were present because those residues are required for the interaction with apoER2 responsible for selenium distribution to extra-hepatic tissues and for protection of brain neurons under selenium-deficient conditions. Also, mutation of the first UGA to UCA resulted in a doubling of Sepp1 forms (mostly the shorter ones) in plasma, probably indicating an increase in mutant Sepp1 synthesis.

### 3.4. Deletion of the second SECIS element

SECIS 2 was deleted to assess the effect of its absence on plasma Sepp1 and on selenium transport to tissues. Homozygous SECIS 2 deletion mice appeared healthy and had no obvious clinical abnormalities. They tolerated severe selenium deficiency, caused by feeding a selenium-deficient diet for 36 weeks after weaning, without developing neurological signs (data not shown).

Plasma from homozygous SECIS 2 deletion mice contained only 15% as much N-terminal Sepp1 as did plasma from WT littermates (figure 5*b*) and plasma Gpx activity was 76% that of the WT littermates (not shown). In spite of the very low Sepp1 and the decrease in Gpx activity, plasma selenium was 58% of that in WT littermates. The liver selenium concentration in the SECIS 2 deletion mice was 113% that of WT littermates but selenium concentrations in other tissues and the whole body were not significantly different from those of WT littermates (not shown).

The Sepp1 N-terminal forms migrated in one band on the SDS-PAGE gel (figure 6, lane 3). That band corresponded to the upper band of WT plasma (lane 1). Purified Sepp1 from SECIS 2 deletion mice contained an average of 7.2 atoms of selenium per molecule (table 1).

These findings indicate that deletion of SECIS 2 leads to a sharp decrease in total plasma Sepp1 forms and a shift to long-form Sepp1 molecules. The decrease in plasma Gpx activity is consistent with a decrease in short form Sepp1, which supplies selenium to the kidney.

### 3.5. Deletion of the first SECIS element

**Figure 7.**
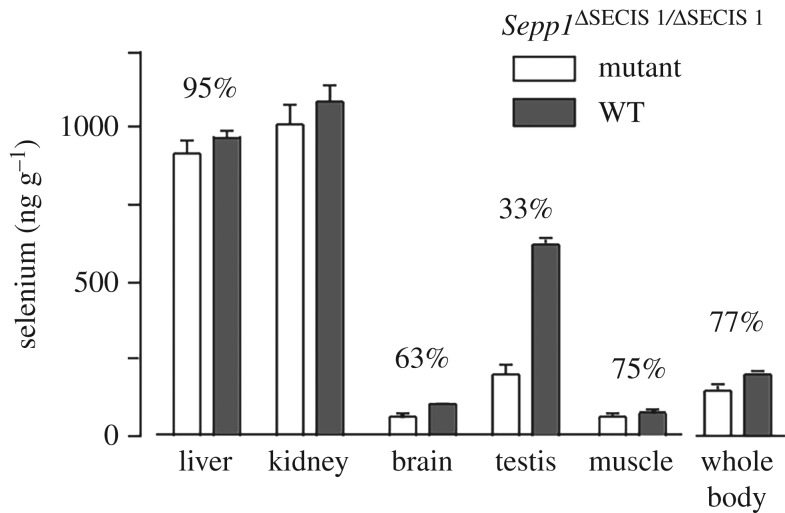
Effects of deleting SECIS 1 element on tissue selenium concentrations. Mice were fed control diet from weaning and were studied four weeks after weaning. Values are means + 1 s.d., *n* = 5. Pairs of values with percentages above them are different (*p* < 0.05) by Student's *t*-test.

Deletion of the first SECIS element had major effects on plasma Sepp1 and on transport of selenium from the liver to peripheral tissues. The plasma selenium concentration was 26% of the WT value, although Sepp1 concentration fell only to 75% (figure 5*c*) and Gpx activity was not affected (not shown). Tissue concentrations of selenium were decreased in the tissues that depend on apoER2-mediated endocytosis of long forms of Sepp1 for their supply of selenium (figure 7). Kidney selenium, which depends on megalin-mediated endocytosis of short form Sepp1 for its selenium, was not affected.

Homozygous SECIS 1 deletion mice did not tolerate selenium deficiency. Four such male weanling mice were fed a selenium-deficient diet and four were fed the same diet supplemented with 0.25 mg selenium per kilogram (control diet). None of the mice fed the selenium-deficient diet survived beyond four weeks, whereas all those fed the control diet appeared healthy. One selenium-deficient mouse was sacrificed at two weeks because it had severe impairment of gait and had also developed hyperactivity. Another one had lost 31% of its body weight by two weeks and was sacrificed. A third mouse became severely hyperactive and was sacrificed at three weeks and the fourth developed gait impairment at two weeks that progressed; it was found dead at four weeks (table 2).

**Table 2.** Summary of findings in selenoprotein P homozygous mouse strains.

	WT	*Δ*1–361	*Δ*240–361	U40S	ΔSECIS 2	ΔSECIS 1
Plasma selenoprotein P	control	0%	145%	200%	15%	75%
plasma Se^a^	control	18%	47%	66%	58%	26%
plasma Gpx^a^	control	100%	100%	100%	76%	100%
Se atoms/selenoprotein P	5^b^	—	1.0 (mass spec)	1.6	7.3	1.0
tissue Se^a^	control	decreased in tissues dependent on apoER2	decreased in tissues dependent on apoER2	no effect	no effect	decreased in tissues dependent on apoER2
forms on gel^a^	mostly long with some short	—	only short	increased short	only long	increased short
neurological injury with 0 Se	no	yes	yes	no	no	yes

^a^Compared with WT littermates.

^b^Plasma biomarkers are from unpublished results and other findings are from Hill *et al.* [47].

In a separate experiment, four weanling female homozygous SECIS 1 deletion mice were fed a selenium-deficient diet and three were fed a control diet. Three of the mice fed the selenium-deficient diet developed neurological signs that were less severe than those seen in the males. One was sacrificed at four weeks and the other two at five weeks. The fourth mouse fed a selenium-deficient diet remained without neurological signs at five weeks, as did the three mice fed the control diet. The neurological impairment that was seen in males and in females was typical of that seen in homozygous Sepp1 deletion mice fed a selenium-deficient diet [69].

The Sepp1 fraction obtained from the SECIS 1 deletion mouse plasma was subjected to SDS-PAGE in a separate experiment and the result is shown in figure 6, lane 4. The dominant protein is at the 67 kDa position and migrates with albumin. The predominant product migrating slower than the 50 kDa position appears at the 37 kDa position instead of just below 50 kDa, where long forms of Sepp1 would be expected. An even less dense band is visible below that reflecting the 37 kDa product. The presence of significant protein contamination, as indicated by the 67 kDa protein, is compatible with very low Sepp1 amounts being in the 2 µg sample loaded. A possible explanation is that the plasma contained very low amounts of Sepp1 forms that bound 9S4.

The one preparation available for selenium analysis contained an average of 1.0 selenium atom per molecule (table 1). Because the Sepp1 preparation from the SECIS 1 deletion strain contained more contaminants than the other preparations (figure 6, compare lane 4 with lanes 1–3), its Sepp1 likely contains more than 1.0 selenium atom per molecule.

In summary, deletion of SECIS 1 had striking effects. It caused a sharp decrease in plasma Sepp1 and also in selenium concentration of tissues expressing apoER2, but not in kidney, which expresses megalin. This strongly suggests that it drastically decreased long-form Sepp1 while having a lesser effect on short-form Sepp1.

## 4. Discussion

### 4.1. Isoform diversity

Exploration of the mechanism of decoding multiple UGAs as selenocysteine has been accompanied by identification here of previously unrecognized diversity in selenoprotein P mRNAs.

Further to polyA-Seq analysis showing alternative forms of Sepp1 mRNA, it is notable that one human Sepp1 mRNA variant has been found to lack SECIS 2. This shorter form of human Sepp1 mRNA that lacks SECIS 2 is present in lower abundance than the canonical form, and interestingly no difference in the ratio in different tissues has been detected. The only prior report of a eukaryotic selenoprotein mRNA lacking a SECIS was selenoprotein S [21] and in that case it was the sole SECIS element. With polyA-Seq data [59], we confirmed the presence of distal polyA sites consistent with the SelS SECIS-lacking variant mRNA (electronic supplementary material, figure S2A). However, our analysis of these data did not reveal the tissue specificity we separately detected with GTEx data where the isoform was detected at low levels in all tissues, but it showed a high expression peak in the testis samples. Instead proportionate expression of the SECIS-lacking and the canonical form of SelS mRNA appears to be roughly the same across the samples from different tissues, including testis-derived samples (electronic supplementary material, figure S2*b*,*c*). We did not experimentally address whether presumptive regulation of SelS may be linked to some condition such as selenium availability. Future work will need to address potential functional significance of these two forms and extend analysis of the 3′ variant lacking the sole SECIS studied previously [21] and also here.

While much remains unknown about how SECIS elements in the 3′ UTR inform translating ribosomes that UGA in that mRNA is to specify selenocysteine, even less is known about whether it is associated with selenoprotein 3′ poly(A) interacting in a closed-loop arrangement to facilitate re-initiation involving specialized components. While there are precedents in unrelated mRNAs for considering such loops [70–75], it may be pertinent that at least several selenoprotein mRNAs have unusual 5′ cap structures [76] and initiation factors have some level of specific relevance for selenocysteine specification [77]. Whether significant selenoprotein mRNA closed-loop formation occurs is unknown. Such considerations could be relevant to the existence of truncated isoforms.

Because selenoprotein P has at least two functions and has multiple forms it is possible that cells can regulate the forms they produce. The transport function of mammalian selenoprotein P appears to reside in hepatocytes because 90% or more of plasma Sepp1 is produced by them [45]. Most cell types express Sepp1 but the forms they produce have not been characterized.

Monitoring the ratios of the distinct mRNA forms in hepatic carcinoma cells with varying selenium levels revealed unexpected first evidence for Sepp1 multiphasic expression. There is first a bell-shaped distribution of Sepp1 expression, with a second point of increase at high selenium concentration. A multiphasic distribution was not reported in older papers reporting similar experiments [66]. However, that work involved northern blots and development since then of the more sensitive qPCR used in this work is probably relevant to this difference. The sharp fluctuation of transcript levels also seems to be very sensitive to changes in concentration, and so a fine range of concentrations is also likely important.

More recent studies performed in rodents using qPCR revealed increasing Sepp1 mRNA in response to selenium concentrations which then plateaus off at super-nutritional levels tested, eight times fold higher than required (0.08, 0.24, 0.8. 2 and 5 µg g^–1^ of body weight) [78]. The concentration range tested in our experiments is within normal levels required in humans with maximum values enough to induce toxicity (0, 1, 2, 5, 10, 20, 50 and 100 nM). The mRNA expression for all the transcript variants start to drop at selenium levels considered optimum (10–20 nM) and a late increase was further observed at toxic concentration (100 nM). It is possible that in the rodent studies, key concentrations required to display the multiphasic response are missed. Another important point to note is that underlying selenium regulation is further complicated due to an often huge discrepancy between selenium metabolism in intact animals versus cultured cell models. Future work will need to assess the generality of the initial observation reported here, and address the possibility that in addition to the known role of Sepp1 in selenium transport to different tissues, at very high selenium levels Sepp1 levels may again increase but now to facilitate excretion and detoxification.

Although no evidence for selenium concentration dependent alteration of isoform ratio was obtained, future experiments may reveal stress or other conditions in which ratio alteration occurs and has functional significance (Sepp1 reduces oxidative stress *in vivo* by an unknown mechanism). Though WT mice lack a natural isoform with SECIS 1 but without SECIS 2, we generated a mouse in which the sole Sepp1 gene lacked SECIS 2 and analysed its phenotype when fed different levels of selenium in its food.

### 4.2. SECIS and U40S mutant mice

The mice in which the second SECIS element was deleted had only 15% as many molecules of Sepp1 in their plasma as WT but it was almost exclusively ‘longform’. As used above, ‘longform’ includes the apoER2 binding site specified by codons 324–326 and is distinct from the short forms synthesized by ribosomes that do not extend to codon 326. As UGA 5 is codon 311, this long-form Sepp1 normally has multiple selenocysteines. Under standard cage conditions, these mice had normal tissue selenium levels and tolerated selenium deficiency without neurological injury. Determination of the likely significance of the newly identified human isoform lacking SECIS 2 will require considerable future work.

**Figure 8.**
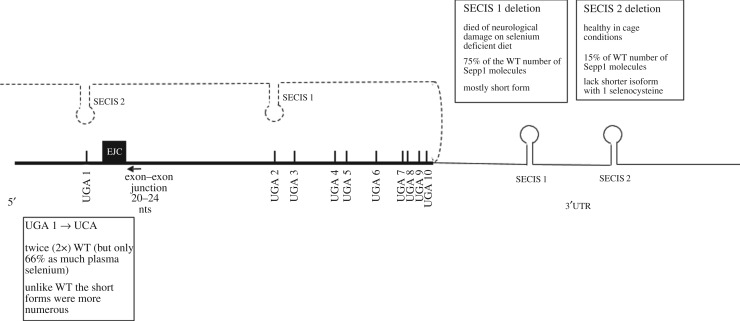
Sepp1 mRNA and summary of mutant mice findings. Schematics of Sepp1 mRNA including relative positions of UGA 1–10, exon junction complex located 20–26 nucleotides 3′ of UGA 1 and SECIS elements in the 3′ UTR. Dashed line represents 3′ UTR with SECIS elements looping as in the Berry model [33]. The positioning of SECIS 2 and SECIS 1 is intended to illustrate their prime role in facilitating UGA 1, and UGA 2–10, respectively, in specifying selenocysteine.

Plasma Sepp1 was analysed earlier from WT mice fed a selenium-deficient diet [79,80]. The efficiency of UGA redefinition is modulated by selenium availability [37], as is the expression of several selenoprotein mRNAs. Since only low levels of plasma Sepp1 are seen in mice fed on a selenium-deficient diet, the Sepp1 analysis for presentation here was only performed on mice fed a selenium-replete diet. Also relevant is that especially at low selenium concentrations, the selenocysteine tRNA and the SECIS machinery can cause UGA to specify cysteine [41,81]. Sepp1 is synthesized and processed inside cells before being secreted into the plasma. Our observations (figure 8) were made on extracellular protein and, therefore, reflect transcriptional, mRNA processing and stability influences on mRNA levels as well as translation, protein processing and export. As selenocysteine specification is in competition with standard decoding in which UGA is a stop codon, mRNA decay is especially relevant. There is a conserved exon–intron junction 3′ of UGA 1; it is only 26 nt distant and so well under the 50 nt minimal spacing ‘rule’ required for canonical nonsense-mediated decay susceptibility. The level of Sepp1 mRNA is lower in selenium-deprived conditions than in replete conditions, but its proportionate increase is much less than that of its glutathione peroxidase 1 and selW counterparts which are also much more sensitive to nonsense-mediated decay [24,82–86]. Major aspects on nonsense-mediated decay effects remain to be resolved and are outside the scope of the present work as are other types of mRNA decay, notably No-go decay [25], which could be potentially relevant when ribosomes pause at UGA. Though SECIS-binding protein 2 binds Sepp1 less avidly than many other selenoprotein mRNAs [84], it plays a major role in protecting Sepp1 mRNA from decay [31,46]. (It also binds to SECIS elements [26], preferentially to SECIS 1 [84].) All three mutants studied here show less than a 25% difference in Sepp1 mRNA levels from their WT counterpart (data not shown). Because there are many Sepp1 forms, it is possible that some of those translated might not survive processing, by the ER and Golgi apparatus, to be secreted. However, the findings do show that SECIS 1 and SECIS 2 have different effects on enabling ribosomes to decode UGA as selenocysteine, and that either the location in the mRNA or whether the UGA is the first, second or third in relation to the other UGAs, affects the efficiency of selenocysteine specification.

In mice with UGA 1 and SECIS 2 present but with SECIS 1 deleted, there is a 25% reduction of plasma Sepp1 from that of WT. The decrease may be due to processing or differences in turnover. Alternatively, it could reflect the proportion of SECIS 1-mediated selenocysteine specification at UGA 1 in WT. That SECIS 1 must be able to act at UGA 1 in the absence of SECIS 2 is shown by the presence in the SECIS 2 deletion mice of 15% of the amount of Sepp 1 present in WT mice. Nearly this entire product is present in long form with implications for different SECIS 1 action at downstream UGAs than occurs with WT mRNA. We strongly favour the interpretation that SECIS 1-mediated selenocysteine specification at UGA 1 leads to greater ribosome specialization at that site than occurs with SECIS 2. The results also suggest that most of the Sepp1 product in plasma in WT mice derives from SECIS 2 rather than SECIS 1 being involved in decoding UGA 1, with SECIS 1 normally being involved in decoding subsequent UGAs. Thus, the present results broadly support the Berry model for SECIS 1 and SECIS 2 roles derived from the very different approach of directly assaying Sepp1 from zebra fish Sepp1 sequences fused to GST and expressed in transiently transfected mammalian cells [13]. *In vitro* translation in both rabbit reticulocyte lysates [14] and in wheat germ lysates [15] also broadly supported the Berry model.

One deduction from the earlier construct-based work was that Sec incorporation at the first UGA codon is inefficient, whereas Sec incorporation at downstream UGAs occurs with increased efficiency. Our SECIS 1 and SECIS 2 mutant studies also support this, and the results from mice with UGA 1, codon 40, substituted with a serine codon (U40S) are also relevant. With U40S, all ribosomes would be expected to progress to codon 240 where they would encounter their first UGA (the position of UGA 2 in WT). The amount of plasma Sepp1 was twice as much as in WT. Presumably on reaching UGA 1 some ribosomes terminate instead of incorporating selenocysteine and continuing translation. The unknown proportion of such early terminating ribosomes may be relevant to elevated Sepp1 with U40S, though possible slow decoding of UGA 1 could also be pertinent if it leads to a ribosome pile-up that could affect initiation. If indeed a substantial proportion of ribosome specialization occurs at UGA 1 in WT, UGA 1 could be slower to decode than downstream UGAs. However, additionally in U40S mice, even with both SECIS elements present, most of plasma Sepp1 was present in truncated forms (short forms). While the products may conceivably be more stable and so more detectable than their WT counterparts, it seems more likely that the absence of UGA 1 location-specific ribosome specialization is the cause of the disproportionate increase of short forms compared with full-length forms. (Notably, however, mice engineered to express only the N-terminal domain, with deletion of codons 240–361 and so SECIS 1 not involved in decoding UGAs after UGA 1, had a Sepp1 plasma level of 145% of the WT level [47]. Because the reason(s) for this is unknown, it is unclear whether it is relevant to interpreting the U40S results, but the possibility of mRNA structural effects cannot be excluded.)

Prior *in vitro* studies showed greater processivity after an initial restrictive event [14]. Though nothing is known of the decoding of the 28 UGA codons in sea urchin selenoprotein P mRNA, it is tempting to imagine that decoding of multiple distal UGAs is processive without involvement of independent SECIS interaction events. In mice with their Sepp1 mRNA lacking SECIS 1, consistent with ribosome specialization and its more effective mediation by SECIS 1, the detectable plasma products mediated by SECIS 2 action are almost all short form, likely due to termination at UGAs 2 or 3. To detect termination at UGA 1, we would have had to use an upstream reporter and this could have affected native upstream influences on the process. SECIS 2-mediated ribosome specialization at UGA 2 and downstream UGAs are at best ineffective. The difference between the two SECIS elements could be due to any, or a combination, of several reasons. One is potential differences in *trans*-acting components associated with the two SECIS elements. Another is different responses to factors at or close to, UGA 1. Permissible mRNA folding could also be relevant. Finally, SECIS 1 but not SECIS 2 may stably track with the specialized ribosomes.

The finding of mostly truncated Sepp1 with the UGA 1 substitution mutation points to events at UGA 1 being important for the ribosome specialization (figure 8). How does the complex of the SECIS element involved and its associated proteins, know where and how to contact the ribosome to ‘inform’ it that the first UGA is a selenocysteine codon and not to mediate termination? It has been suggested that the involvement of SECIS 2, but not SECIS 1, would be more readily explicable if the former had features of their spatially restrictive class of SECIS elements [57]. This may be one of the relevant features, but others merit consideration. Our preliminary data show no hints that a particular nascent peptide signal interaction mediated ribosomal conformation change may be relevant. If a small guide RNA has a role, perhaps directing mRNA base modification, then it lurks undetected. However, an exon junction complex might be very relevant to the properties of the UGA 1 substitution mutant. The exon junction protein eIF4a3, which shares homology to initiation factor eIF4a1, is a selective negative regulator of selenoprotein synthesis. During selenium deficiency, expression of eIF4a3 is upregulated several fold and binds to a variable extent to type 1 SECIS elements preventing, where tested, Sec insertion [27,28]. Whether the hypermethylated cap structure of selenoprotein mRNAs obviating eIF4E in initiation [76] is relevant is unknown. eIF4E is normally loaded onto mRNA with eIF4G that tethers the 43S ribosome complex and also interacts with polyA binding protein. When eIF4G is itself tethered to the 3′ UTR of dicistronic mRNAs, it was found to stimulate translation of the second ORF probably by RNA looping [75]. Competition for its likely binding to SECIS 2 (a type 1 element) by SBP2 is presumably facilitated by weak binding of SBP2 to SECIS 2. This binding is likely weaker than SECIS 1 [84], raising the potential for a tethering role for linking the SECIS-associated complex to the vicinity of UGA 1. However, while SBP2 is really important for Sepp1 expression [29,30], it is not essential and intriguingly plays a major role in stabilizing Sepp1 mRNA [31]. Whether the intron junction is relevant to this is unknown. As noted above, the distance, 26 nt, from UGA 1 in pre-mRNA to a downstream intron is highly conserved. After splicing, the exon junction complex, which spans 20–24 nt 5′ of the exon–exon junction and contains eIF4a3, will be very close to the first UGA. Could the potential tethering role of eIF4a3 at this exon junction complex to SECIS 2 (possibly with involvement of L30) mediate a coupling to a ribosome approaching UGA 1 with subsequent disruption of the eIF4a3 interaction being relevant to loading of other factors, including EFsec, before the UGA enters the ribosomal A site? While the presence of this intron in a highly conserved position is likely relevant to decoding of UGA 1, it is clearly not an absolute requirement since constructs lacking this intron can still recode the first UGA (although high levels of termination were also observed and the constructs used had a reporter upstream rather than the WT sequence arrangement [13]).

The conclusion that though SECIS 1 can suffice, the presence of SECIS 2 substantially enhances selenocysteine specification at UGA 1 raises the question as to why SECIS 2 was selected. Is there some reason why SECIS 1 was not selected to do the whole task? One possibility is that selection for high processivity of SECIS 1 proved incompatible with a counter selection for ribosome specialization at UGA 1 that generated a certain level of short-form Sepp1. SECIS 2 may have been the result.

### 4.3. Contrasting effects of features close to mRNA 3′ ends on UGA redefinition

The major role of SECIS element(s) in the 3′ UTR of eukaryotic selenoprotein mRNAs in mediating the recoding of UGA to specify selenocysteine contrasts with strong evidence for features of the mRNA 3′ ends being necessary for UGA, and also UAA and UAG, to specify termination in ciliates [87–89]. While in some ciliates occurrences of one or more of these codons in internal regions of coding sequences is at least mostly avoided, in at least one species all 64 specify universal amino acids [87,88]. Relevance to the present work comes from the emerging evidence that even in mammalian cells natural termination has more components than previously appreciated, and includes some level of mRNA 3′ end associated features [90].

### 4.4. Origin of selenoprotein P

The mammalian selenoprotein P gene (named Sepp1, or also SelP, SelPa or Sepp1a) emerged early in vertebrates, by duplication of a gene that encoded only its current N-terminal domain. This shorter gene, named Sepp1b (or SelPb), is still present in the genome of fishes, birds, reptiles and non-placental mammals, while it was lost in placentals [91]. Sepp1b mRNA contains a single Sec-specifying UGA and a single SECIS. It appears that extended vertebrate selenoprotein P first appeared with a very Sec-rich C-terminus (more than 15 residues), and then the number of Sec residues was reduced in many terrestrial tetrapods, including human and mouse, by conversion to cysteine codons [42]. The gene duplication originating selenoprotein P has characteristics consistent with its occurrence within one of the reported rounds of whole-genome duplications at the root of vertebrates [92]. Specifically, we predict the duplication to have occurred in early Gnathostomata (jawed vertebrates) after the split with Cyclostomata (jawless fishes). In fact, the genome of sea lamprey *Petromyzon marinus* contains the parental Sepp1b gene, but not the selenoprotein P gene (electronic supplementary material, figure S3*b*). Sea urchin also possess a selenoprotein P gene with multiple Sec residues [42]; however, this appears to have emerged in a distinct phylogenetic event. Further research is currently ongoing to better characterize the evolution of selenoprotein P outside vertebrates. Throughout the duplication that generated Sepp1, intron positions were conserved, so that they are shared by the extant vertebrate selenoprotein P and SelPb genes. Thus, the intron next to UGA 1 is ancestral, already present in the parental gene prior to duplication. It is possible, yet speculative, that the presence of the intron at this position played a role in the origin of the selenoprotein P gene itself. We may imagine that in its early phases after duplication, the new selenoprotein P gene could easily harbour multiple UGAs, but probably contained a single SECIS. The hypothesized tethering role of eIF4a3 could have helped deliver the SECIS to the first UGA, which became increasingly distant as the C-terminus tail grew bigger. Although likely inefficient, such a system could have been enough to guarantee a minimum amount of selenoprotein P with multiple Sec residues. Natural selection for this function would then have led to the origin of SECIS 2, likely accompanied by changes to the ancestral SECIS and establishment of the current system.

The appearance of extended vertebrate selenoprotein P apparently coincided both with the emergence of its C-terminus tail and of SECIS 2 in its mRNA; we could not observe any species in intermediate states, e.g. a long selenoprotein P coding sequence with no SECIS 2, or a short coding sequence with both SECIS elements. We attempted to determine the phylogenetic relationship of SECIS elements of selenoprotein P and SelPa genes (see the electronic supplementary material, S3). The SECIS elements analysed cluster in three distinct groups: SECIS 1 of Sepp1, SECIS 2 of Sepp1 and SECIS of Sepp1b. This is consistent with SECIS 1 and 2 being generated by duplication of the SECIS in the ancestor Sepp1b. There is no clear correspondence between the SECIS of Sepp1b and any particular SECIS of selenoprotein P. The sequence identity between the SECIS of Sepp1b and those of selenoprotein P mRNAs are approximately the same. The SECIS in Sepp1b genes is a type 2 element. Interestingly, both selenoprotein P SECIS 1 and 2 are type 2 in fishes, while tetrapods mutated SECIS 2 to type 1 (electronic supplementary material, figure S3*c*).

The results presented here indicate that altering the functions of SECIS 1 or SECIS 2 can alter the forms of Sepp1 protein synthesized by the cell. Perhaps use of regulated SECIS inclusion to modulate the relative and absolute abundance of Sepp1 protein isoforms has been a major driving force in development of the remarkable recoding event involved.

## Supplementary Material

Expression Analysis of Selenoprotein P

## Supplementary Material

Expression analysis of Selenoprotein S

## Supplementary Material

Analysis of SECIS elements in Selenoprotein P genes

## Supplementary Material

Tables of Primers for Selenoprotein P

## Data accessibility

The datasets supporting this article have been uploaded as part of the electronic supplementary material.
