# Supplementary material for: Human selenoprotein P and S variant mRNAs with different numbers of SECIS elements and inferences from mutant mice of the roles of multiple SECIS elements
Source: Open Biol. 2016 Nov 23;6(11):160241. doi: 10.1098/rsob.160241 (PMC5133445; doi:10.1098/rsob.160241)
Supplement: Expression Analysis of Selenoprotein P [file rsob160241supp1.pdf]

Supplementary Material S1

Expression analysis of Sepp1

Legends:

Figure S1A:

Schema of Sepp1 gene structure, including the exons and junctions that were quantified in GTEx. The junctions corresponding to the canonical, most abundant isoform are in bold black. The last exon is not quantified in GTEx due to its overlap with the gene CCDC152.

Figure S1B

Sepp1 exon quantification in GTEx samples, split by subtissue. Exons belonging to the canonical isoform are framed in black rectangles. Non-canonical, minor expression exons are highlighted in colored rectangles. Exons considered under the detection limit are shaded in white. The dotted red lines represent an arbitrary detection limit, an expression threshold of 0.1 RPKM.

Figure S1C

Sepp1 exon junction quantification in GTEx samples, split by subtissue. Junctions belonging to the canonical isoform are framed in black rectangles. Non-canonical, minor expression junctions are highlighted in colored rectangles. Junctions considered under the detection limit are shaded in white. The dotted red lines represent an arbitrary detection limit, an expression threshold of 0.1 RPKM.

Figure S1D

Poly A sites in Sepp1 gene across five mammals. The poly A sites mapping to the 3' UTR region of Sepp1 genes were obtained from (Derti et al., 2012). The read counts were normalized by the total number of reads in each sample, and then aggregated for all samples in each species. The major poly A site was found downstream the SECIS2 (region highlighted in blue) in all species, and a minor poly A site between the two SECIS was identified in human and rhesus macaque. The distance is represented in nucleotides from the Sepp1 stop codon, TAA in all species. Sepp1 gene predictions were obtained using Selenoprofiles (Mariotti and Guigo, 2010) and the SECIS elements were identified with SECIsearch3 (Mariotti et al., 2013).

Figure S1A:

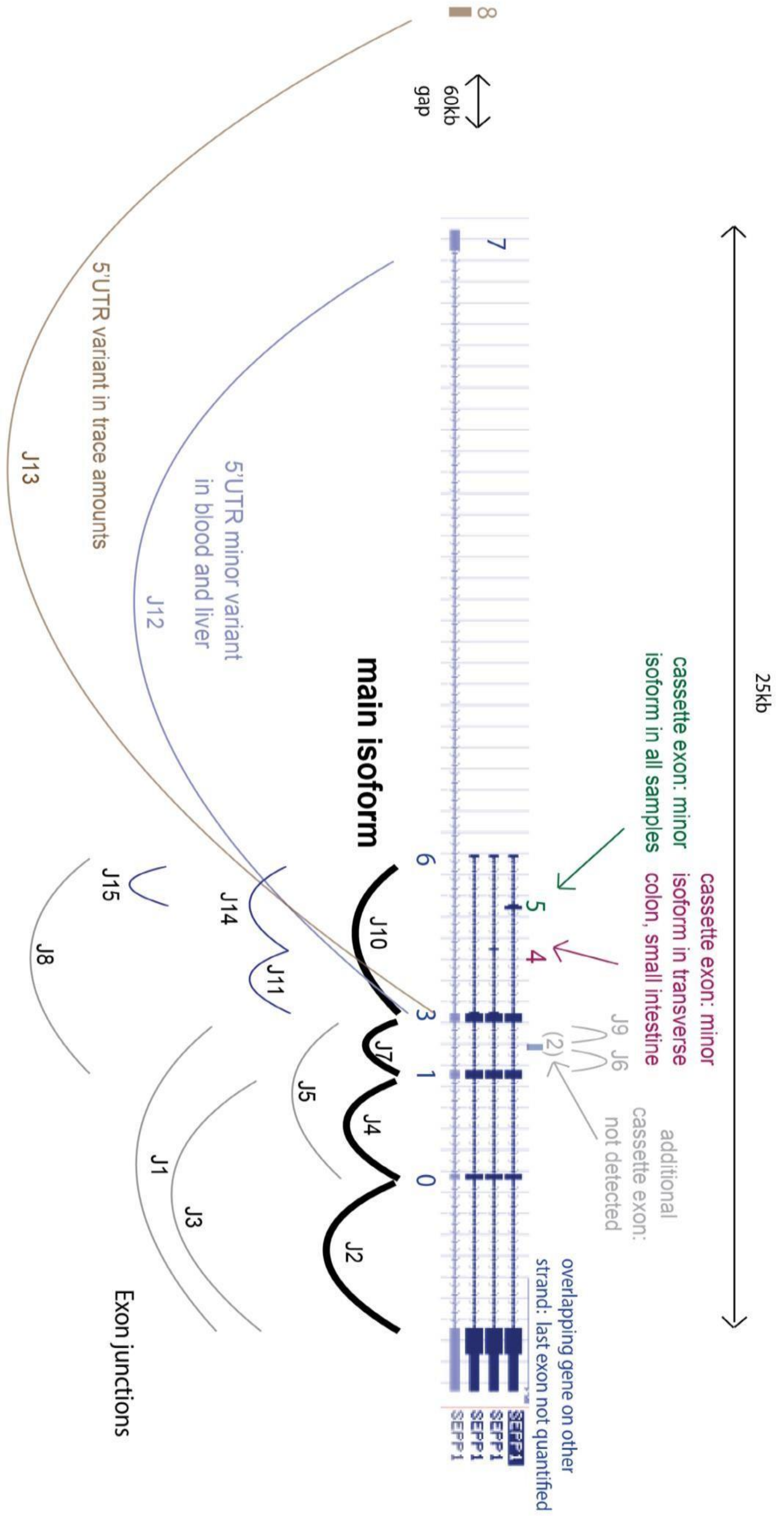

8
60kb gap
25kb
7
5'UTR variant in trace amounts
J13
5'UTR minor variant in blood and liver
J12
cassette exon: minor isoform in all samples
main isoform
cassette exon: minor isoform in transverse colon, small intestine
6
5
4
3
1
0
J9 J6
(2)
additional cassette exon: not detected
overlapping gene on other strand: last exon not quantified
J10
J14
J15
J11
J8
J7
J5
J4
J1
J3
J2
Exon junctions
SEPP1
SEPP1
SEPP1
SEPP1

Figure S1B

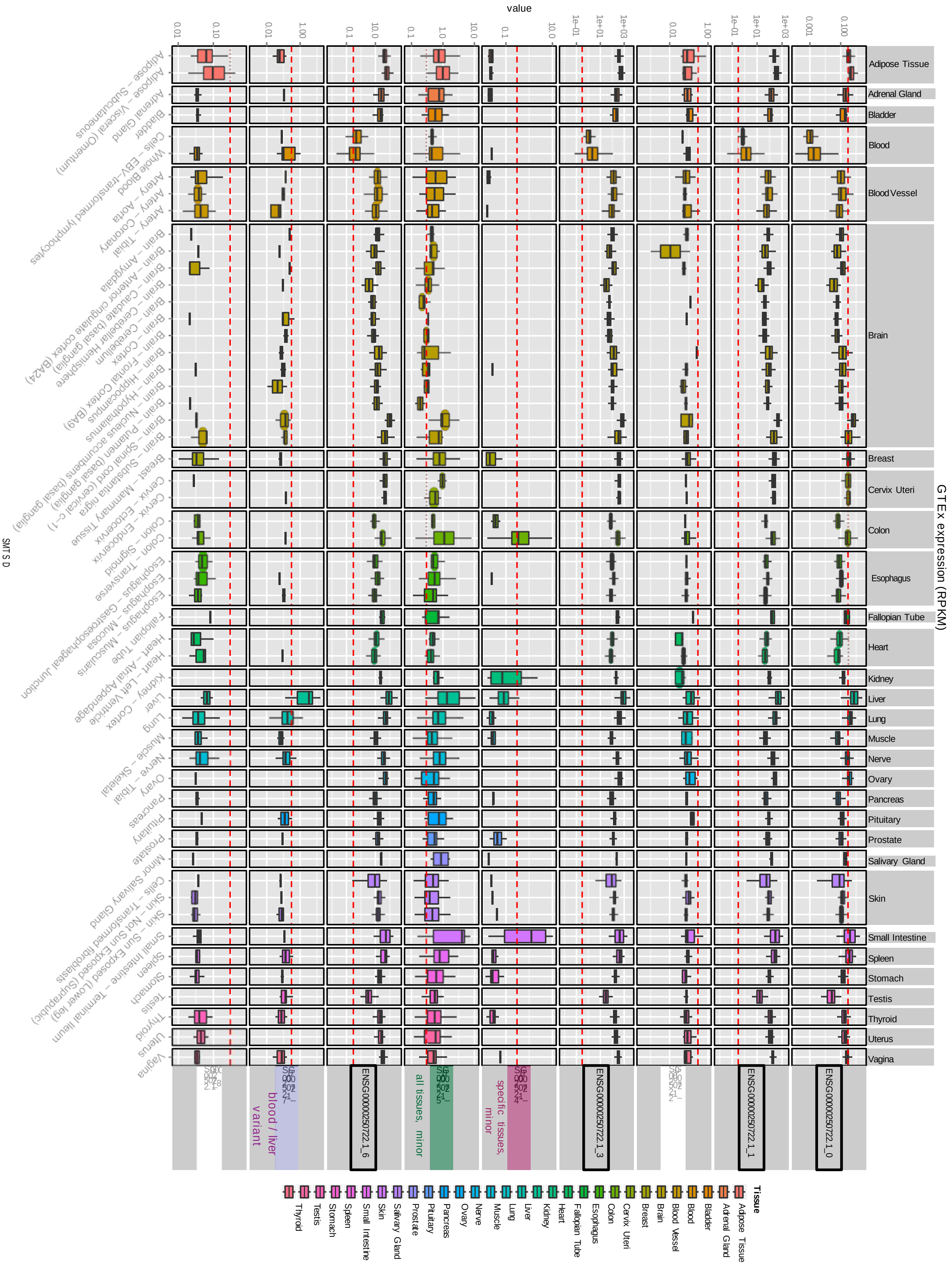

value
GTEx expression (RPKM)
SMTSD
Adipose Tissue
Adrenal Gland
Bladder
Blood
Blood Vessel
Brain
Breast
Cervix Uteri
Colon
Esophagus
Fallopian Tube
Heart
Kidney
Liver
Lung
Muscle
Nerve
Ovary
Pancreas
Pituitary
Prostate
Salivary Gland
Skin
Small Intestine
Spleen
Stomach
Testis
Thyroid
Uterus
Vagina
ENSG00000250722.1_6
ENSG00000250722.1_3
ENSG00000250722.1_1
ENSG00000250722.1_0
blood / liver variant
all tissues, minor
specific tissues, minor
Tissue

Figure S1C

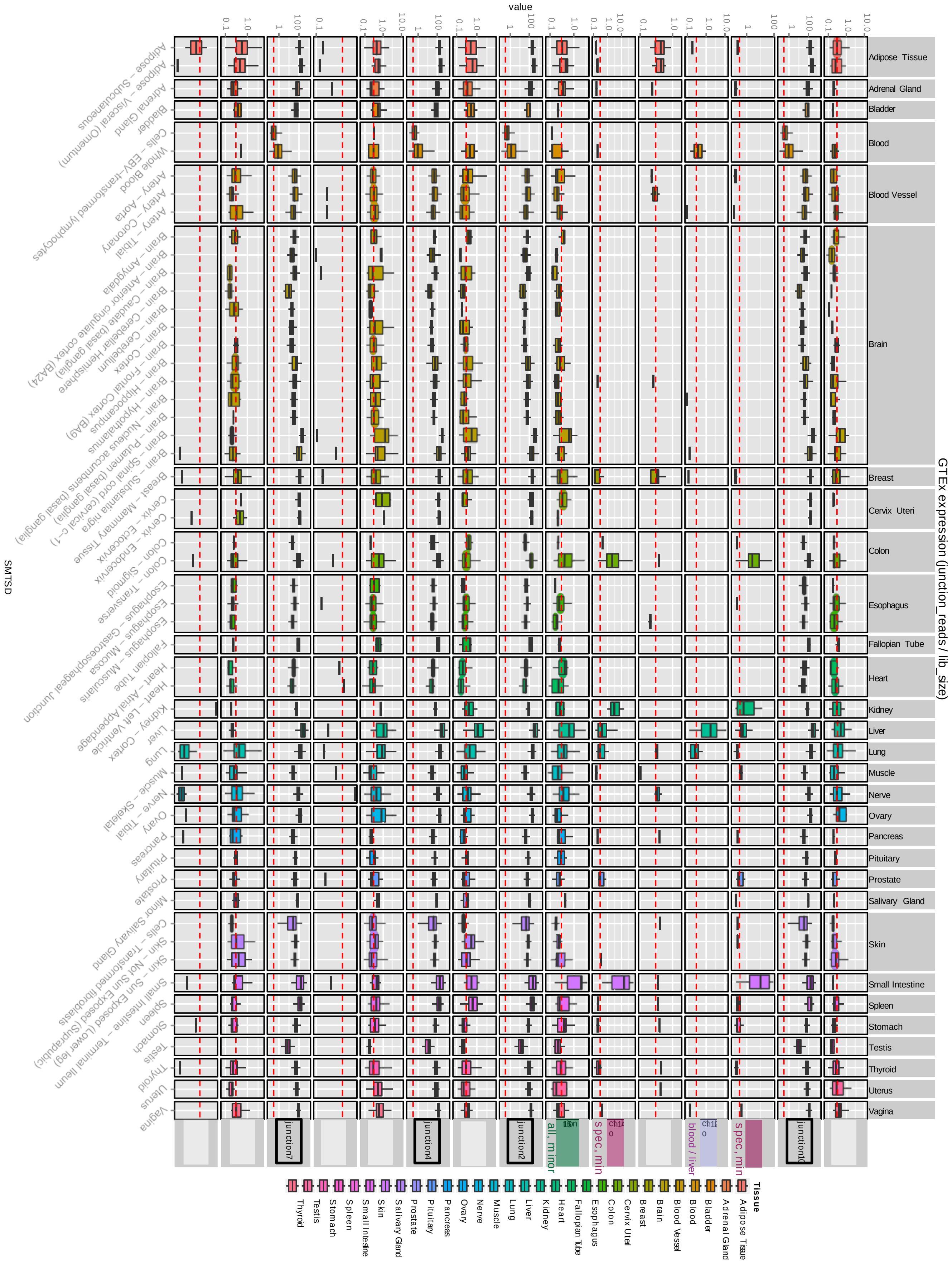

value
SMTSD
GTEx expression (junction_reads / lib_size)
Adipose Tissue
Adrenal Gland
Bladder
Blood
Blood Vessel
Brain
Breast
Cervix Uteri
Colon
Esophagus
Fallopian Tube
Heart
Kidney
Liver
Lung
Muscle
Nerve
Ovary
Pancreas
Pituitary
Prostate
Salivary Gland
Skin
Small Intestine
Spleen
Stomach
Testis
Thyroid
Uterus
Vagina
junction7
junction4
junction2
junction10
all, minor
spec, min
blood / liver
Tissue

Figure S1D:

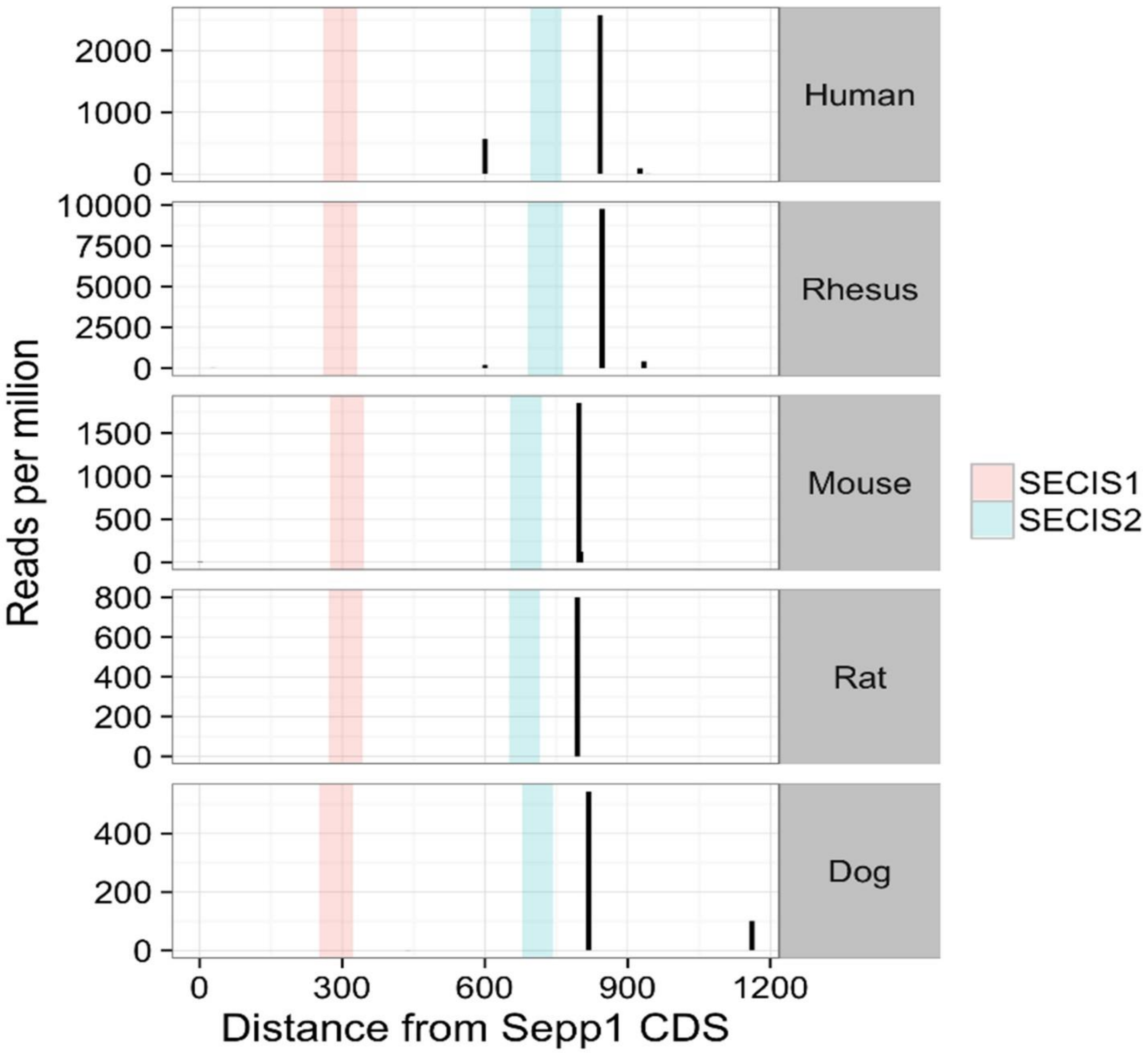

Human
Rhesus
Mouse
Rat
Dog
2000
1000
0
10000
7500
5000
2500
0
1500
1000
500
0
800
600
400
200
0
400
200
0
Reads per milion
0
300
600
900
1200
Distance from Sepp1 CDS
SECIS1
SECIS2
