# Supplementary material for: Human selenoprotein P and S variant mRNAs with different numbers of SECIS elements and inferences from mutant mice of the roles of multiple SECIS elements
Source: Open Biol. 2016 Nov 23;6(11):160241. doi: 10.1098/rsob.160241 (PMC5133445; doi:10.1098/rsob.160241)
Supplement: Expression analysis of Selenoprotein S [file rsob160241supp2.pdf]

Supplementary Material S2

Expression analysis of SelS

Legends:

Figure S2A:

Schema of SelS gene structure (gene name: VIMP), including the exons and junctions that were quantified in GTEx. The exons and junctions that do not belong to the canonical, most abundant isoform are highlighted.

Figure S2B:

SelS exon quantification in GTEx samples, split by subtissue. Non-canonical exons are highlighted. The dotted red lines represent an arbitrary detection limit, an expression threshold of 0.1 RPKM.

Figure S2C:

SelS exon junction quantification in GTEx samples, split by subtissue. Non-canonical exons are highlighted. The dotted red lines represent an arbitrary detection limit, an expression threshold of 0.1 RPKM.

Figure S2A:

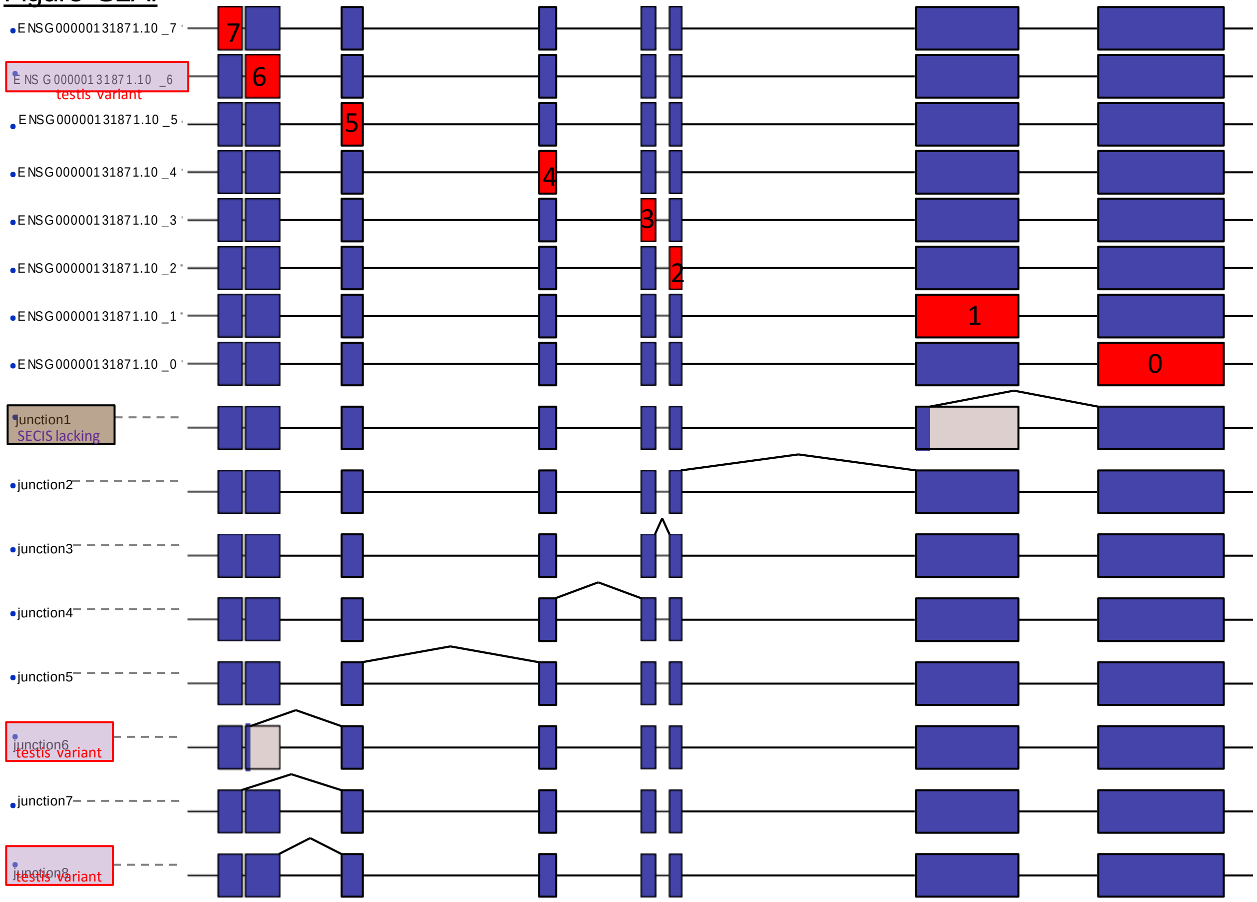

ENSG00000131871.10 _7
ENSG00000131871.10 _6
testis variant
ENSG00000131871.10 _5
ENSG00000131871.10 _4
ENSG00000131871.10 _3
ENSG00000131871.10 _2
ENSG00000131871.10 _1
ENSG00000131871.10 _0
junction1
SECIS lacking
junction2
junction3
junction4
junction5
junction6
testis variant
junction7
junction8
testis variant
7
6
5
4
3
2
1
0

Figure S2B

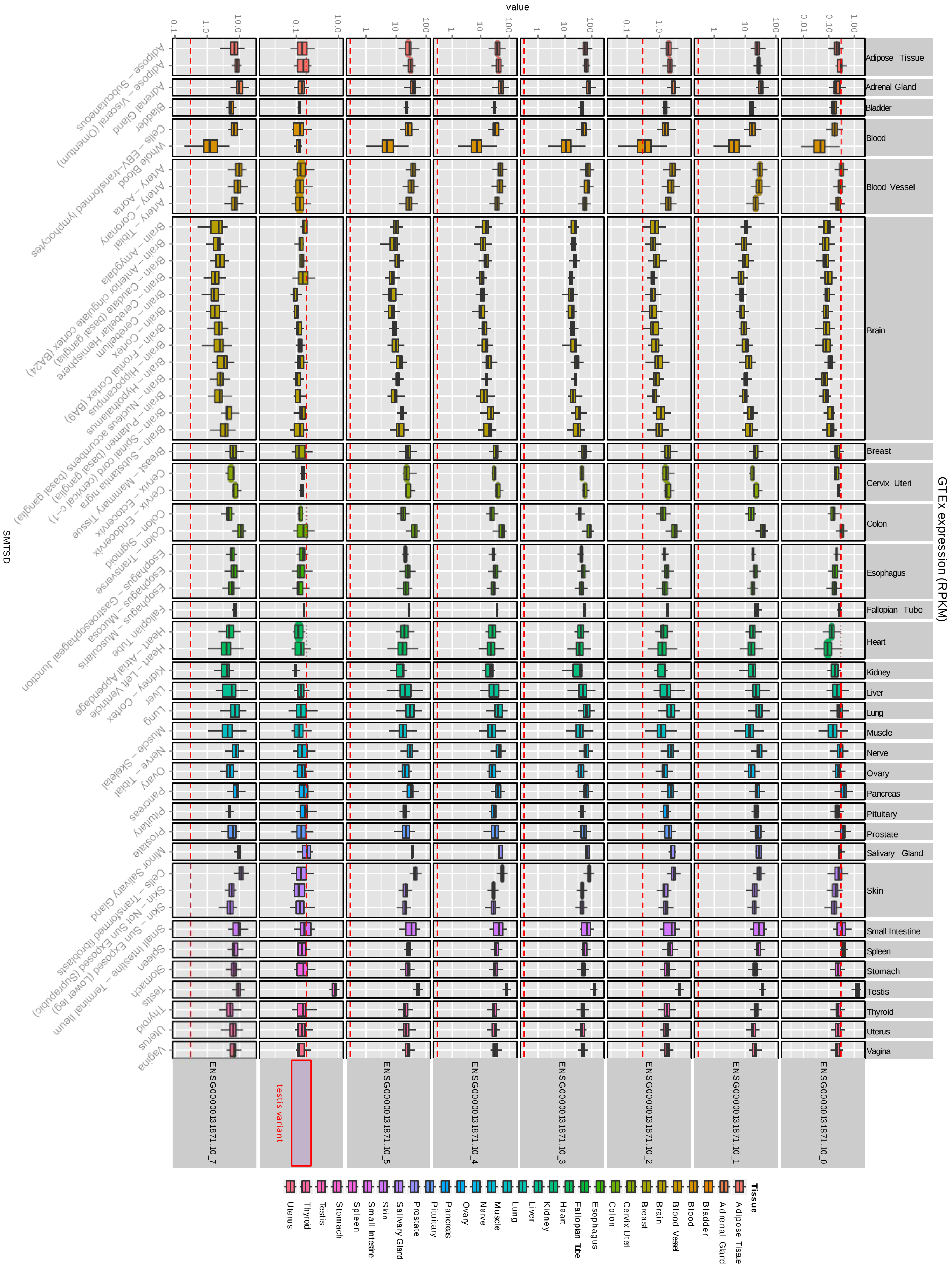

value
GTEx expression (RPKM)
SMTSD
testis variant
ENSG00000131871.10_7
ENSG00000131871.10_5
ENSG00000131871.10_4
ENSG00000131871.10_3
ENSG00000131871.10_2
ENSG00000131871.10_1
ENSG00000131871.10_0
Adipose Tissue
Adrenal Gland
Bladder
Blood
Blood Vessel
Brain
Breast
Cervix Uteri
Colon
Esophagus
Fallopian Tube
Heart
Kidney
Liver
Lung
Muscle
Nerve
Ovary
Pancreas
Pituitary
Prostate
Salivary Gland
Skin
Small Intestine
Spleen
Stomach
Testis
Thyroid
Uterus
Vagina
Tissue

Figure S2C

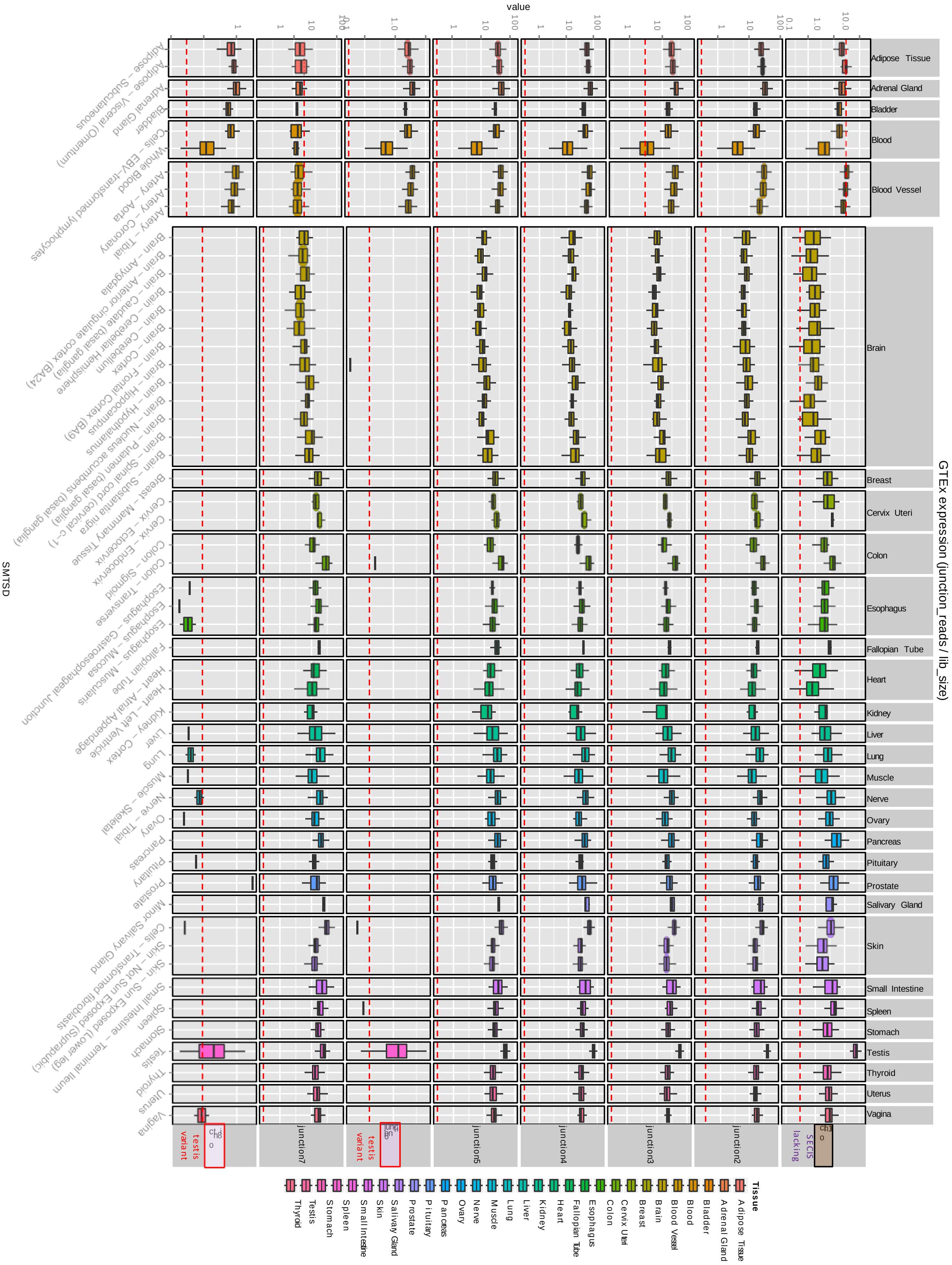
value
GTEx expression (junction_reads / lib_size)
SMTSD
junction7
junction5
junction4
junction3
junction2
testis variant
SECIS lacking
Adipose Tissue
Adrenal Gland
Bladder
Blood
Blood Vessel
Brain
Breast
Cervix Uteri
Colon
Esophagus
Fallopian Tube
Heart
Kidney
Liver
Lung
Muscle
Nerve
Ovary
Pancreas
Pituitary
Prostate
Salivary Gland
Skin
Small Intestine
Spleen
Stomach
Testis
Thyroid
Uterus
Vagina
Tissue
