# Supplementary material for: Human selenoprotein P and S variant mRNAs with different numbers of SECIS elements and inferences from mutant mice of the roles of multiple SECIS elements
Source: Open Biol. 2016 Nov 23;6(11):160241. doi: 10.1098/rsob.160241 (PMC5133445; doi:10.1098/rsob.160241)
Supplement: Analysis of SECIS elements in Selenoprotein P genes [file rsob160241supp3.pdf]

Supplementary Material S3

Analysis of SECIS elements in Sepp1 genes

Legends:

Figure S3A

Alignment of SECIS elements in selenoprotein P genes of representative vertebrate species. SelPa is used to designate the direct homologue of human Sepp1, with multiple in-frame UGAs, while SelPb designates the additional single-UGA containing gene found in non placental vertebrates (Sepp1b). S1 designates the first SECIS found downstream of the coding sequence. S2 designates the second one e.g. SelPa.S1.Alligator_mississipiensis is the first sequence in the multi-Sec Sepp1 of the alligator. The consensus sequence is displayed at the bottom.

Figure S3B

Average distance tree computed by Jalview based on the alignment shown in panel

Figure S3C

Structure of SECIS elements as predicted by SECISearch3 webserver

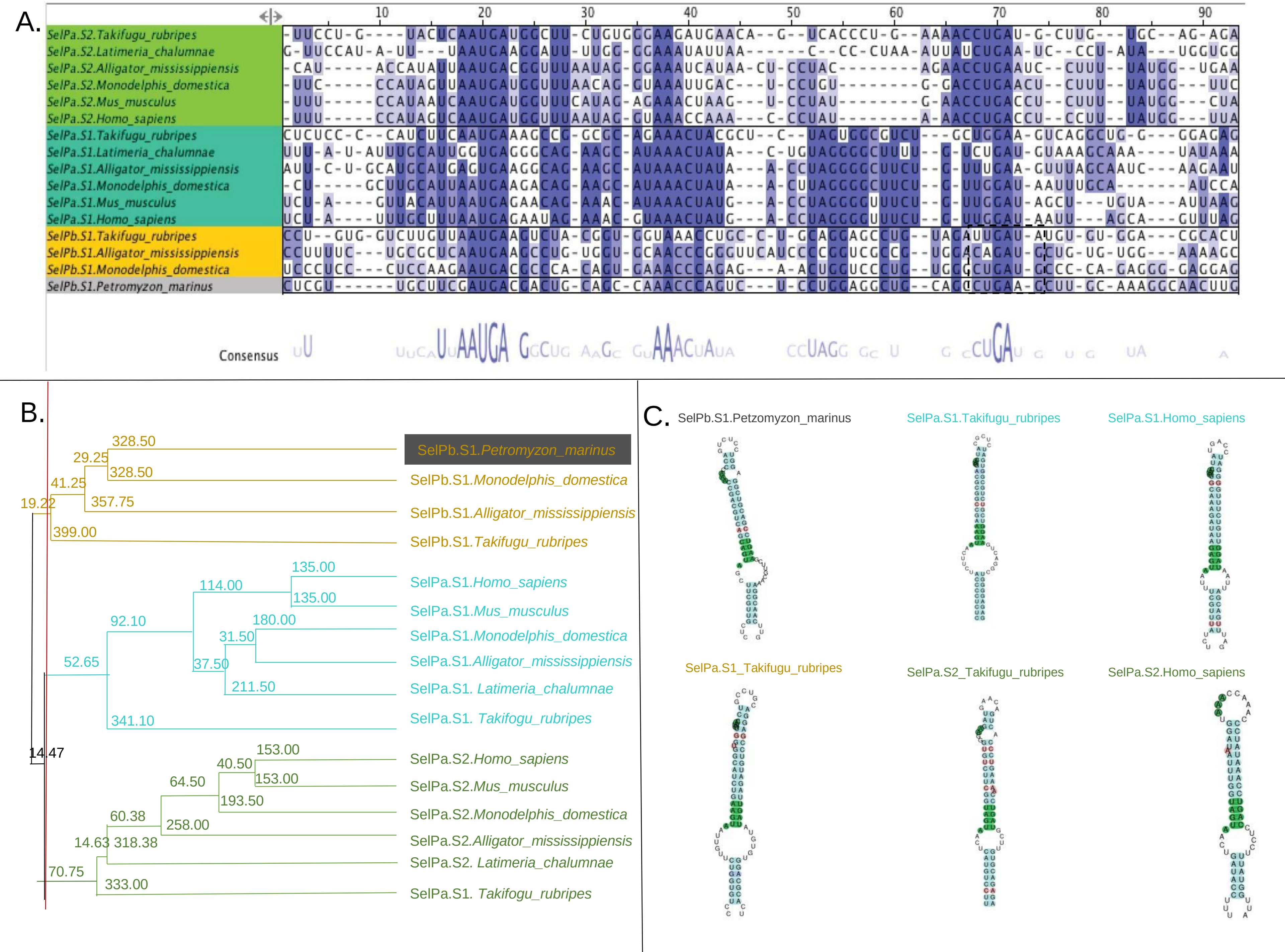
A.
SelPa.S2.Takifugu_rubripes
SelPa.S2.Latimeria_chalumnae
SelPa.S2.Alligator_mississippiensis
SelPa.S2.Monodelphis_domestica
SelPa.S2.Mus_musculus
SelPa.S2.Homo_sapiens
SelPa.S1.Takifugu_rubripes
SelPa.S1.Latimeria_chalumnae
SelPa.S1.Alligator_mississippiensis
SelPa.S1.Monodelphis_domestica
SelPa.S1.Mus_musculus
SelPa.S1.Homo_sapiens
SelPb.S1.Takifugu_rubripes
SelPb.S1.Alligator_mississippiensis
SelPb.S1.Monodelphis_domestica
SelPb.S1.Petromyzon_marinus
Consensus
B.
328.50
29.25
328.50
41.25
19.22
357.75
399.00
SelPb.S1.Petromyzon_marinus
SelPb.S1.Monodelphis_domestica
SelPb.S1.Alligator_mississippiensis
SelPb.S1.Takifugu_rubripes
135.00
114.00
135.00
92.10
180.00
31.50
52.65
37.50
211.50
341.10
SelPa.S1.Homo_sapiens
SelPa.S1.Mus_musculus
SelPa.S1.Monodelphis_domestica
SelPa.S1.Alligator_mississippiensis
SelPa.S1. Latimeria_chalumnae
SelPa.S1. Takifogu_rubripes
14.47
153.00
40.50
64.50
153.00
193.50
60.38
258.00
14.63
318.38
70.75
333.00
SelPa.S2.Homo_sapiens
SelPa.S2.Mus_musculus
SelPa.S2.Monodelphis_domestica
SelPa.S2.Alligator_mississippiensis
SelPa.S2. Latimeria_chalumnae
SelPa.S1. Takifogu_rubripes
C.
SelPb.S1.Petzomyzon_marinus
SelPa.S1.Takifugu_rubripes
SelPa.S1.Homo_sapiens
SelPa.S1_Takifugu_rubripes
SelPa.S2_Takifugu_rubripes
SelPa.S2.Homo_sapiens
