# Supplementary material for: Human selenoprotein P and S variant mRNAs with different numbers of SECIS elements and inferences from mutant mice of the roles of multiple SECIS elements
Source: Open Biol. 2016 Nov 23;6(11):160241. doi: 10.1098/rsob.160241 (PMC5133445; doi:10.1098/rsob.160241)
Supplement: Tables of Primers for Selenoprotein P [file rsob160241supp4.docx]

Supplementary Tables S1

Primer Sequences for Sepp1

Table S1A

Sequences of forward and reverse primers targeting different regions of human SePP for transcript quantification.

| Primer Name | Forward Primer Sequence (5’-3’) | Reverse Primer Sequence (5’-3’) |
| --- | --- | --- |
| A1 | ACGTAAACTATGACCTAGGGGTTTC | TGGAAAGCATGTCTTTGTTGTTCTT |
| A2 | TGACCTAGGGGTTTCTGTTGGA | TCACGTGTTAAGTATTTCTGGATCT |
| B1 | ACTCTTCTAAAACTTGAGTGGCTGT | GGAGGTCAGGTTTATAGGGTTTGGT |
| B2 | TCTTCTAAAACTTGAGTGGCTGTCT | AGGAGGTCAGGTTTATAGGGTTTGG |
| C1 | GGAGCTGCCAGAGTAAAGCA | ACATTGCTGGGGTTGTCAC |
| GAPDH | AGCCTCCCGCTTCGCTCTCT | CCAGGCGCCCAATACGACCA |

Table S1B

Primers used for generation of mouse line

| Primer Name | Primer sequence |
| --- | --- |
| WS785 | GTAGTGCTACTTATAAATCCCAACAGAGTCTCTTTTAGCTGTTGCTTCAGCGACTGAATT GGTTCCTTTAAAGCC |
| WS786 | ATGTTTATTGCTCACCTAGGCATTATACTAAACACTCCATGCAAACTACAGCCGCACTCG  AGATATCTAGACCCA |
| WS789 | GAAAGCGTGGTGTTAATCTGCGTACTGCTTAAGACAGTATTTCCATAATCAGGatCCAGC ATTACACGTCTTGAGCGATTGT |
| WS790 | CACTTAATTCTGTACTCCATTCTTGCTTAGTCGTATTAGCCATAAAAGAGGatCCCACTTA  ACGGCTGACATGGGAATTA |
| WS869 | GAGATCCCAGTAGGTAGGCGACTTG |
| WS870 | CCTGTAACCTTGAGCCAAACTTCCT |
| WS871Sepp1--3F | AGATAAGAGAATTGTTAACCAAATTTGTTGGGTGAGCCTA |
| WS872Sepp1  -3R | cacaatGATGTGCTTTGCACAGTCTAGGACA |
| WS1214SECI  S1camF | CAATAGGGCACCTGAAAAGTGACTGCAGCCTTTGGTTAATATGTCTTTCTTGGATCCAG CATTACACGTCTTGAGCGATTGT |
| WS1215SECI  S1camR | CAGAGTTTAAGTAAAGAAAAAAAAAACTGGAAAATATGTCTTTGTTGTTCGGatCCCAC TTAACGGCTGACATGGGAATTA |
